# The P2X7 Receptor in Inflammatory Diseases: Angel or Demon?

**DOI:** 10.3389/fphar.2018.00052

**Published:** 2018-02-06

**Authors:** Luiz E. B. Savio, Paola de Andrade Mello, Cleide Gonçalves da Silva, Robson Coutinho-Silva

**Affiliations:** ^1^Laboratory of Immunophysiology, Biophysics Institute Carlos Chagas Filho, Federal University of Rio de Janeiro, Rio de Janeiro, Brazil; ^2^Division of Gastroenterology, Department of Medicine, Beth Israel Deaconess Medical Center, Harvard Medical School, Harvard University, Boston, MA, United States; ^3^Division of Vascular Surgery, Department of Surgery, Center for Vascular Biology Research, Beth Israel Deaconess Medical Center, Harvard Medical School, Boston, MA, United States

**Keywords:** purinergic signaling, extracellular ATP, inflammation, macrophages, lymphocytes, neurodegenerative diseases, inflammatory disease, sepsis

## Abstract

Under physiological conditions, adenosine triphosphate (ATP) is present at low levels in the extracellular milieu, being massively released by stressed or dying cells. Once outside the cells, ATP and related nucleotides/nucleoside generated by ectonucleotidases mediate a high evolutionary conserved signaling system: the purinergic signaling, which is involved in a variety of pathological conditions, including inflammatory diseases. Extracellular ATP has been considered an endogenous adjuvant that can initiate inflammation by acting as a danger signal through the activation of purinergic type 2 receptors—P2 receptors (P2Y G-protein coupled receptors and P2X ligand-gated ion channels). Among the P2 receptors, the P2X7 receptor is the most extensively studied from an immunological perspective, being involved in both innate and adaptive immune responses. P2X7 receptor activation induces large-scale ATP release via its intrinsic ability to form a membrane pore or in association with pannexin hemichannels, boosting purinergic signaling. ATP acting via P2X7 receptor is the second signal to the inflammasome activation, inducing both maturation and release of pro-inflammatory cytokines, such as IL-1β and IL-18, and the production of reactive nitrogen and oxygen species. Furthermore, the P2X7 receptor is involved in caspases activation, as well as in apoptosis induction. During adaptive immune response, P2X7 receptor modulates the balance between the generation of T helper type 17 (Th17) and T regulatory (Treg) lymphocytes. Therefore, this receptor is involved in several inflammatory pathological conditions. In infectious diseases and cancer, P2X7 receptor can have different and contrasting effects, being an angel or a demon depending on its level of activation, cell studied, type of pathogen, and severity of infection. In neuroinflammatory and neurodegenerative diseases, P2X7 upregulation and function appears to contribute to disease progression. In this review, we deeply discuss P2X7 receptor dual function and its pharmacological modulation in the context of different pathologies, and we also highlight the P2X7 receptor as a potential target to treat inflammatory related diseases.

## Introduction

Adenosine triphosphate (ATP) has long been known as the intracellular energy currency molecule of the cell. Under physiological conditions, extracellular ATP (eATP) is present at low levels (nanomolar range). However, this phosphate compound can be released by stressed, injured, or dying cells, reaching high concentrations (hundred micromolar) in the extracellular milieu (Pellegatti et al., 2008; Wilhelm et al., 2010). Once outside the cells, ATP and its metabolites (ADP, AMP, and adenosine) generated by the action of ecto-enzymes—named ectonucleotidases—mediate a high evolutionary conserved signaling system: the Purinergic Signaling (Burnstock and Verkhratsky, 2009; Verkhratsky and Burnstock, 2014).

Purinergic Signaling was first reported in the 1920s by Drury and Szent-Gyorgyi, when the effects of adenine compounds in the circulatory system of mammals were described (Drury and Szent-Gyorgyi, 1929). In the 1950s, studies showed that ATP was released from sensory nerves (Holton and Holton, 1954; Holton, 1959), but only in the 1970s, adenine compounds were recognized as signaling molecules thanks to Geoffrey Burnstock's studies (Burnstock et al., 1970, 1972; Burnstock, 1972). Interestingly, the first article reporting the effects of eATP in immune cells also dates back to the 1970s (Dahlquist and Diamant, 1970). In their paper, Dahlquist and Diamant (1970) described that eATP induces histamine release from mast cells. Further studies involving these same cells have led to the discovery of a specific receptor for eATP, later identified as the P2X7 receptor subtype (Cockcroft and Gomperts, 1980). Since then, especially in the last three decades, eATP-P2X7 receptor signaling has become one of the most studied pathways in infectious and inflammatory diseases.

P2X7 receptor is a ligand-gated ion channel belonging to the purinergic type 2 receptor family (P2). P2 receptor family comprises the P2Y G protein–coupled receptors (P2Y_1,2,4,6,11−14_) and P2X receptors (P2X1–7), which are ligand-gated ion channels (Ralevic and Burnstock, 1998; Abbracchio et al., 2006). P2X7 is the most extensively studied receptor subtype from an immunological perspective. Its sustained stimulation by millimolar concentrations of eATP, triggers non-selective pore formation, which allows the passage of molecules of up to 900 Da, Na^+^ and Ca^2+^ influx and K^+^ efflux resulting in changes in the ionic homeostasis of the cell (Coutinho-Silva and Persechini, 1997). In addition, P2X7 receptor can initiate the release of large-scale intracellular ATP via its intrinsic pore formation ability or in association with pannexin hemichannels, therefore boosting purinergic signaling and inflammation (Pelegrin and Surprenant, 2006).

Several other functions have been attributed to P2X7 receptor in innate and adaptive immune responses. It is widely expressed by different immune cells including monocytes, macrophages, neutrophils, lymphocytes, mast cells, among others (reviewed in Lenertz et al., 2011; Jacob et al., 2013; Idzko et al., 2014; Morandini et al., 2014b). During innate immune response, damage-associated molecular patterns (DAMPs) or pathogen-associated molecular pattern (PAMPs) activate pattern recognition receptors (PRRs) (i.e., Toll-like receptors—TLRs) inducing ATP release, which in turn can activate P2X7 receptor (Cohen et al., 2013). In addition, TLR-mediated NF-κB pathway activation act as the first signal promoting the transcription of several genes encoding inflammatory mediators including pro-IL-1β and inflammasome components, such as NLRP3 and ASC. P2X7 receptor stimulation represents the second signal to inflammasome activation by triggering K^+^ efflux, inflammasome assembly, and subsequent caspase-1 activation. The later, in turn, processes pro-IL-1β to its mature form which is able to be released then (Ferrari et al., 1997, 2006; Qu et al., 2007; Ting et al., 2008; Di Virgilio et al., 2017). P2X7 receptor also promotes IL-6 release in a Ca^2+^-dependent mechanism (Shieh et al., 2014). Moreover, P2X7 receptor stimulates the production of free radicals (Cruz et al., 2007; Hewinson and Mackenzie, 2007; Hung et al., 2013) and it is involved in the activation of caspases and phospholipases (Coutinho-Silva et al., 2003b; Kahlenberg et al., 2005; Costa-Junior et al., 2011), as well as in cell cycle regulation and apoptosis (Coutinho-Silva et al., 1999; Bianco et al., 2006). P2X7 receptor also modulates intracellular signaling pathways, such as MyD88/NF-κB, PI3K/Akt/mTOR, and the activation of mitogen-activated protein kinase (MAPK) pathway proteins (MEK, ERK 1/2) (Bradford and Soltoff, 2002; Skaper et al., 2010; Liu et al., 2011; Bian et al., 2013; Savio et al., 2017a).

During adaptive immune response, P2X7 receptor is directly involved in T cell activation. Indeed, ATP-P2X7 receptor signaling is required for TCR-mediated calcium influx and IL-2 production. The blockading of P2X7 receptor-mediated calcium influx inhibited T cell activation (Yip et al., 2009). Moreover, P2X7 receptor modulates the balance between the generation of T helper type 17 (Th17) and T regulatory (Treg) lymphocytes (Schenk et al., 2011; Cekic and Linden, 2016). ATP-P2X7 receptor signaling decreases the suppressive activity and viability of Treg cells and favors the polarization of T cells into Th17 cells (Schenk et al., 2011). In addition, P2X7 receptor's blockade facilitates the conversion of naive CD4^+^ T cells into Treg cells (Schenk et al., 2011).

Taking into account its crucial role in immune response, it is expected that an imbalance in P2X7 receptor activation may favor several pathological conditions including infectious, inflammatory, and neurodegenerative diseases, as well as cancer. Up until now, several studies have pointed different and contrasting effects for P2X7 receptor, whose activation may be able to either potentiate or ameliorate disease progression. In this review, we discuss P2X7 receptor's dual function and its pharmacological modulation in the context of different pathologies, as well as highlight its potential use as a therapeutic target for the treatment of inflammatory related diseases.

## P2X7 receptor agonists, antagonists, and knockout mice—important considerations

To date, no specific agonist for P2X7 receptor has been described (De Marchi et al., 2016). The endogenous P2X7 receptor ligand, eATP itself, has distinct effects on P2X7 activation depending on its concentration at the active site (Steinberg and Silverstein, 1987; Virginio et al., 1999; Hibell et al., 2000). As a rule, high eATP concentrations (in the millimolar range—EC50≥ 100 μM) are required to activate P2X7 receptor (Surprenant et al., 1996; Bianchi et al., 1999; Donnelly-Roberts et al., 2009). While high micromolar levels of ATP stimulates P2X7 receptor to form a cation-selective channel, prolonged exposure to millimolar levels trigger a nonselective large cytolytic pore conformation, allowing the passage of 900 Da molecules through the plasma membrane (Steinberg and Silverstein, 1987; Virginio et al., 1999; North, 2002). eATP is readily metabolized by extracellular ectonucleotidases, dropping initially high eATP levels to much lower levels (nanomolar to low micromolar), wherein it can activate other P2X subtypes (De Marchi et al., 2016). In this context, the non-hydrolyzable derivative ATP substrate, ATPγS, is a better alternative for studies involving P2 receptor activation. The benzoyl ester of ATP, BzATP, is far the most potent P2X7 agonist available, being ~10–30 times more potent than ATP—with a low micromolar EC50 for the human receptor (Surprenant et al., 1996; Bianchi et al., 1999). However, it can also activate other P2X subtypes (such as P2X1 and P2X3) and it is metabolized to other adenine derivatives (De Marchi et al., 2016). Besides lack of specificity, differences in agonist potency across mammalian species are also an aggravating factor for studies involving P2X7 receptor (Hibell et al., 2000).

Several molecules have been developed to block P2X7 receptor activity (De Marchi et al., 2016). They can be subdivided into orthosteric ligands—binding the receptor within the ATP-binding cavity—and allosteric ligands—binding the receptor at sites, other than the ATP-binding cavity, and decreasing the effect of the endogenous ligand ATP (De Marchi et al., 2016). The first group is represented by suramin or suramin-like derivatives, ATP derivatives (TNP-ATP, periodate-oxidized ATP [oATP]), tetrazole derivatives (A438079, A839977), and cyanoguanidine derivatives (A740003, A804598) (De Marchi et al., 2016). Among them, tetrazole and cyanoguanidine derivatives present the highest potency and selectivity for P2X7 receptor vs. other P2X and P2Y receptors (Honore et al., 2006; Nelson et al., 2006; Carroll et al., 2007; Donnelly-Roberts et al., 2009; Adinolfi et al., 2015; Amoroso et al., 2015). Their IC50 values vary according to the compound and the mammalian species: A438079 IC50 is 0.13 and 0.32 μM at the human and rat P2X7 receptors (Nelson et al., 2006; Donnelly-Roberts et al., 2009), A839977 IC50 is 0.02–0.150 μM at recombinant human, rat, and mouse P2X7 receptors (Florjancic et al., 2008; Honore et al., 2009; Friedle et al., 2010), A740003 IC50 is 0.040 and 0.020 μM at human and rat P2X7 receptor (Honore et al., 2006; Adinolfi et al., 2015; Amoroso et al., 2015), and A804598 IC50 is 0.0109, 0.0099, and 0.0089 μM at the human, rat, and mouse P2X7 receptors, respectively (Donnelly-Roberts et al., 2009). Unlike the tetrazole and cyanoguanidine derivatives, ATP derivatives (TNP-ATP and oATP) are potent P2X7 receptor antagonist at high micromolar levels and can interact with other P2X receptors (Di Virgilio, 2003; De Marchi et al., 2016). Moreover, besides being an irreversible P2X7 antagonist (Easterbrook-Smith et al., 1976), oATP itself appears to exert anti-inflammatory effects, modulating the immune response independently of P2X7 blockage (Beigi et al., 2003; Di Virgilio, 2003; Figliuolo et al., 2014). In this way, experiments using oATP to evaluate P2X7 role in inflammatory diseases should be carefully analyzed.

The second group of P2X7 blocking molecules is represented by a class of synthetic negative allosteric modulators such as Brilliant Blue G (BBG), AZD9056, KN-62, AZ-11645373, AZ-10606120, GW791343, GSK314181A, GSK1482160, CE-224,535, AFC-5128, JNJ-479655, and EVT-401 (Guile et al., 2009; Friedle et al., 2010; Kaczmarek-Hájek et al., 2012; Alves et al., 2013; North and Jarvis, 2013; Mehta et al., 2014). Even though these compounds present nanomolar/micromolar potency at the P2X7 receptor, they can still interact with other P2X receptors' allosteric binding sites. For example, BBG have been widely used as a selective antagonist for P2X7 receptor, but it can also block P2X1, P2X4, and sodium channels (Jiang et al., 2000; Seyffert et al., 2004; Jo and Bean, 2011). Therefore, divergent and unexpected results found in P2X7 receptor studies might be attributed to different experimental settings where diverse agonist/antagonist drugs with different affinity and specificity were used and hence should be critically analyzed.

Regarding P2X7 receptor knockout (P2X7 KO) mice, at least two strains are currently commercially available. One, generated by GlaxoSmithKline, in which the *lacZ* gene and neomycin cassette (Neo) were inserted into exon 1, and the second, from Pfizer (commercially available from The Jackson Laboratory), which has a Neo insertion in exon 13—exon coding for the long C–terminal cytoplasmic tail (Sikora et al., 1999; Solle et al., 2001). However, the identification of P2X7 splice variants revealed that both knockout mice express P2X7 receptor on T cells, whereas DCs, macrophages, and neurons do not (Taylor et al., 2009; Masin et al., 2012). Although both P2X7 KO mice express P2X7 receptor on T cells, only P2X7 KO mice from GlaxoSmithKline have a functional P2X7 receptor in these cells (Taylor et al., 2009). T cells obtained from Pfizer P2X7 KO mice did not respond to BzATP stimulation, while lymphocytes from GlaxoSmithKline P2X7 KO mice showed high levels of P2X7 activity in comparison to wild type (WT) mice (Taylor et al., 2009).

Taken together, these reports indicate that studies using GlaxoSmithKline KO mice for evaluating P2X7 receptor relevance in an immunological context should be carefully analyzed considering the tissue specific expression of a functional P2X7 protein in T cells.

## P2X7 receptor in infectious diseases—angel or demon depending on the type of pathogen, virulence, and severity of infection

**Figure 1:**
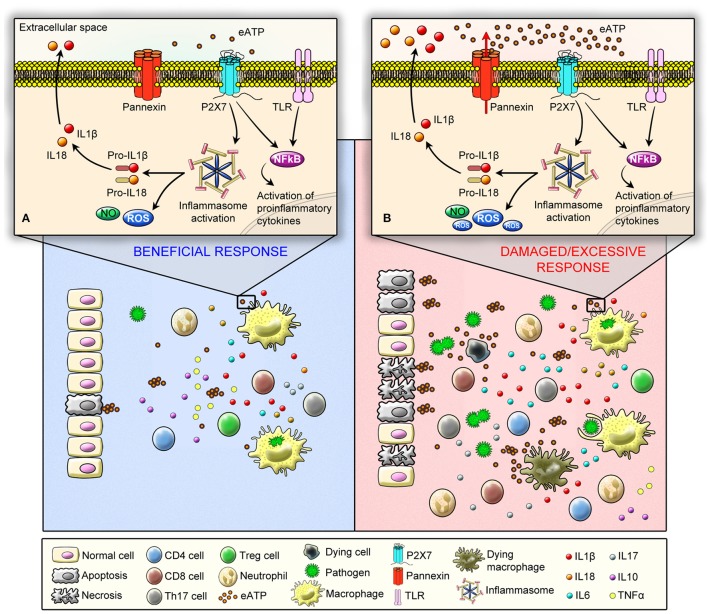
Schematic illustration showing P2X7 receptor protective (angel) and deleterious (demon) effects in immune responses against pathogens. The recognition of pathogen-associated molecular pattern (PAMPs) by Pattern Recognition Receptors (PRRs) can induce ATP release, which activates P2X7 receptor. As a consequence, P2X7 receptor activation induces ATP release—chiefly via pannexin hemichannels—boosting inflammation. **(A)** At a molecular level (upper panel) P2X7 receptor beneficial effects are mediated by the stimulation of microbicidal mechanisms and production of inflammatory mediators in phagocytic cells, such as ROS, NO, and interleukins. P2X7 receptor acts as a second signal for NLRP3 inflammasome activation and IL-1β release. In addition, at a cellular level (low panel) P2X7 receptor is involved in the activation of effector T cells, and it favors the polarization of T cells into Th17 cells and decreases the suppressive activity and viability of Tregs. **(B)** On the other hand, P2X7 can act as a demon depending on the type of pathogen, virulence, and severity of infection by inducing an excessive production and release of inflammatory mediators (upper panel) coupled to a high incidence of apoptotic and necrotic cell death due the release of large amounts of ATP (low panel), which results in sustained P2X7 receptor activation, leading to a self-sustained pro-inflammatory deleterious cycle.

In response to viral, bacterial, fungal, and protozoa infection, ATP is released from immune and non-immune cells. Subsequent activation of the ATP-gated P2X7 receptor has been implicated in the pathophysiology of several infectious diseases through modulation of innate and adaptive immune responses (Coutinho-Silva and Ojcius, 2012; Morandini et al., 2014b; Savio and Coutinho-Silva, 2016; Di Virgilio et al., 2017). Interestingly, P2X7 receptor activation can generate both beneficial and deleterious effects depending on the type of pathogen, virulence, and severity of infection (Figure 1). In the next sections, both positive and negative effects of P2X7 receptor activation are discussed. In addition, the effects of P2X7 receptor pharmacological inhibition or genetic deletion in infectious disease are summarized in Table 1.

**Table 1:** Protective or deleterious effects of P2X7 receptor pharmacological inhibition or genetic deletion in infectious disease.

**Pathogen or PAMP**	**Pharmacological inhibitor or genetic deletion**	**Effect**	**Inflammatory mediator or immune cell involved**	**References**
**VIRAL INFECTIONS**				
Vesicular stomatitis virus	P2X7 KO mice	Deleterious	↓IFN-β ↑viral replication	Zhang et al., 2017
Adenoviral vectors	oATP, A438079, P2X7 KO mice	Protective	↓IL-1β, IL-6, NO, and neutrophil infiltration	Lee et al., 2012
Influenza virus	P2X7 KO mice	Protective	↓INF-γ, TNF-α, IL-6, and neutrophil infiltration	Leyva-Grado et al., 2017
HIV viral proteins in brain cells	oATP, A438079, BBG, suramin	Protective	↓ NF-κB activation, TNF-α, IL-1β, ROS, NO, MCP-1, and neuronal damage	Tewari et al., 2015; Chen et al., 2016
HIV	PPADS, suramin	Protective	↓ HIV-1 fusion CD4^+^ cells	Swartz et al., 2014
HIV	oATP, A740003, BBG, and suramin	Protective	↓ HIV replication in macrophages	Hazleton et al., 2012
**BACTERIAL INFECTIONS**				
*C. trachomatis*	P2X7 KO mice	Deleterious	↓IL-1β ↑ bacterial burden	Darville et al., 2007
*P. gingivali*s	P2X7 KO mice	Deleterious	↓IFN-γ, IL-17	Ramos-Junior et al., 2015
*M. tuberculosis*				
•H37RV	P2X7 KO mice	Deleterious	↑ Treg ↑ bacterial burden	Santos et al., 2013
•Beijing 1471 or MP287/03	P2X7 KO mice	Protective	↓IL-1β and INF-γ ↓ bacterial burden	Amaral et al., 2014
*M. bovis*	P2X7 KO mice	Protective	↓immature-like myeloid cells ↓ bacterial burden	Bomfim et al., 2017
Sepsis				
•Endotoxic shock (LPS)	P2X7 KO mice	Protective	↑ survival ↓cytotoxicity	Yang et al., 2015
•CLP model	A438079, P2X7 KO mice	Protective	↓IL-1β, CXCL1 and CX3CL1 ↑ survival	Wang et al., 2015a
•CLP model	A740003	Protective	↓ p-NF-κB, IL-1β, IL-6,	Wu et al., 2017a
•CLP model	P2X7 KO mice, BBG	Protective	↓p-NF-κB, IL-1β, IL-6, NO, ALT, and neutrophil infiltration ↑ survival	Santana et al., 2015 Savio et al., 2017a Savio et al., 2017b
•CLP model	P2X7 KO mice, oATP	Deleterious	↑ IL-1β, IL-6, TNF-α and bacterial burden ↓ survival	Csóka et al., 2015a
**FUNGAL INFECTIONS**				
*C. albicans*	KN-62	Deleterious	↓DC activation, PGE2	Xu et al., 2016
*P. brasiliensis*	P2X7 KO mice	Deleterious	↓Th17/Th1 response ↑ fungal burden	Feriotti et al., 2017
**PROTOZOA INFECTIONS**				
*L. amazonensis*	P2X7 KO mice, A740003	Deleterious	↓ IL-1β and LTB4	Chaves et al., 2014
*L. amazonensis*	P2X7 KO mice	Deleterious	↑IFN-γ ↓ IL-17, IL-12, and TGF-β ↑parasitic load	Figliuolo et al., 2017a
*T. gondii*	P2X7 KO mice	Deleterious	↓TNF-α ↓survival	Miller et al., 2011
*T. gondii*	P2X7 KO mice	Deleterious	↓IFN-γ, TNF-α, IL-6,CD4^+^ cells ↑parasitic load	Huang et al., 2017
*T. gondii*	P2X7 KO mice	Deleterious	↓ IL-1β, IL-12, TNF-α, and IFN-γ ↑parasitic load	Corrêa et al., 2017
*T. gondii*	P2X7 KO mice	Deleterious	↓ IL-1β and ROS ↑parasitic load	Moreira-Souza et al., 2017
*T. cruzi*	P2X7 KO mice	Deleterious	↑ mast cells	Meuser-Batista et al., 2011
*P. chabaudi*	P2X7 KO mice	Deleterious	↓Th1 response	Salles et al., 2017
*E. histolytica*	oATP, KN-62	Deleterious	↓ IL-1β	Mortimer et al., 2015
**HELMINTH INFECTIONS**				
*H. polygyrus*	BBG	Deleterious	↓Mast cell activation ↓IL-13	Shimokawa et al., 2017
*S. mansoni*	P2X7 KO	Deleterious	↑TGF-β1 ↓ survival	Oliveira et al., 2014

### P2X7 receptor in viral infections

Host macrophages and L929 cells secrete ATP by exocytosis and/or pannexin channels in response to viral infections (Zhang et al., 2017). Extracellular ATP, acting via P2X7 receptor, regulates immune responses against several types of viruses. ATP-P2X7 signaling decreases viral replication and consequently protects bone marrow-derived macrophages (BMDM), macrophage cells line RAW 264.7, and HEK 293T cells from cell death mediated by vesicular stomatitis virus (VSV) infection *in vitro*. This happens by inducing IFN-β secretion via activation of P38/JNK/ATF-2 signaling pathways (Zhang et al., 2017). *In vivo*, ATP treatment reduces viral replication and improves survival of VSV-infected WT mice. This antiviral effect is not observed in P2X7 KO mice (Zhang et al., 2017). Additionally, activation of P2X7 receptor is crucial to control infection of human monocytes by dengue virus-2 (Corrêa et al., 2016). Those findings suggest an important role for this receptor in restraining viral replication and infection. On the other hand, P2X7 receptor activation boosts inflammation and potentially contributes to an exacerbated immune response, depending on the virulence and severity of the infection. In this context, Lee et al. (2012) showed that genetic deletion or pharmacological inhibition of P2X7 receptor improves survival in a mouse model of acute respiratory distress syndrome induced by intranasal administration of replication deficient adenoviral vectors. Improved outcome observed in these settings correlated with decreased production of inflammatory mediators (i.e., IL-1β and IL-6) and reduced neutrophil infiltration (Lee et al., 2012). Similar results were observed in P2X7 KO mice infected with a lethal dose of influenza virus (Leyva-Grado et al., 2017).

A role for extracellular ATP and P2X7 receptor in human immunodeficiency virus (HIV) infection has also been reported (Barat et al., 2008; Swartz et al., 2014, 2015; Graziano et al., 2015). Broad-spectrum P2 receptor antagonists, such as PPADS and suramin, significantly inhibit HIV-1 membrane fusion in CD4^+^ cells (Swartz et al., 2014, 2015). In addition, P2X7 inhibitors oATP, A740003, BBG, and suramin decrease HIV replication in human macrophages (Hazleton et al., 2012). Graziano et al. (2015) showed that ATP induces the release of HIV-1 virions derived from virus containing compartments (subcellular vacuoles), present in monocyte-derived macrophages. P2X7 receptor blockade with A438079 prevented eATP-induced release of virions from monocyte-derived macrophages and D-U1 cells, a chronically HIV-1infected promonocytic cell line. Imipramine, an inhibitor of ceramide formation, also blocked virions release from these cells, suggesting that P2X7 activation stimulates ceramide production, thus favoring the formation of exosomes containing HIV-1 virions. Therefore, targeting P2X7 receptor might be a suitable therapeutic strategy to eliminate HIV-1 reservoirs in individuals receiving combination antiretroviral therapy (cART) (Graziano et al., 2015).

Evidence supporting the involvement of P2X7 receptor in the deleterious effects caused by HIV-1 infection in the central nervous system (CNS) has also been described (Swartz et al., 2015). After primary infection, HIV virus invades the CNS, resulting in neuroinflammation and neurodegeneration. Viral proteins and inflammatory factors produced during the immune response against HIV induce blood-brain barrier (BBB) dysfunction and activate glial cells. BBB dysfunction facilitates transmigration of infected monocytes and CD4^+^ T cells, further propagating infection and inflammation within the CNS. The HIV viral protein called transactivator of transcription (Tat) is described as an important neurotoxin detected in the CNS of HIV-1 infected subjects and it is responsible for triggering P2X7 receptor overexpression in human astrocytes and to promote neuroinflammation (Tewari et al., 2015). Tat enhances the expression of monocyte chemoattractant protein 1 (MCP-1) in human astrocytes in a P2X7-dependent manner (Tewari et al., 2015). In addition, the glycoprotein gp120 (gp120)—an HIV envelope glycoprotein—increases P2X7 receptor expression in BV2 microglia cells (Chen et al., 2016) and in hippocampus of rats (Liu et al., 2017). Pretreatment with BBG has prevented NF-κB activation and production of inflammatory mediators induced by gp120 in BV2 cells (Chen et al., 2016), suggesting a key role for P2X7 receptor in HIV-induced glial cells activation and brain damage.

These findings support the contribution of P2X7 receptor in viral infections promoting protective and deleterious effects depending on viral strain and severity of the infection, which makes the use of P2X7 receptor pharmacological blockers or activators a challenging task on these settings.

### P2X7 receptor in bacterial infections

In the 90's, Humphreys and Dubyak (1996) published one of the first reports describing the enhancement of P2X7 receptor-mediated cell responses, such as membrane permeability and Ca^2+^ influx, in response to lipopolysaccharide (LPS). Currently, it is well-documented that TLRs activation by bacterial products, such as LPS, induces ATP release from immune cells, modulating inflammatory responses (Coutinho-Silva and Ojcius, 2012; Cohen et al., 2013; Morandini et al., 2014b; Di Virgilio et al., 2017). The role of P2X7 receptor in activation of microbicidal mechanisms and production of inflammatory mediators in phagocytic cells, as well as modulation of adaptive immune responses to bacterial infection, have been extensively studied (Coutinho-Silva and Ojcius, 2012; Morandini et al., 2014b; Di Virgilio et al., 2017). In this section, we highlight the role of this purinergic receptor in some important bacterial diseases, including Chlamydia disease, Tuberculosis, Periodontitis, and in bacterial Sepsis.

### Chlamydiae infections

Chlamydiae are obligatory intracellular pathogenic bacteria that infect epithelial cells and macrophages. Chlamydiae can evade host defense mechanisms mainly by inhibiting phagosome–lysosome fusion and acidification (Herweg and Rudel, 2016; Pettengill et al., 2016). Interestingly, ATP, acting through P2X7 receptor, decreases bacterial load in macrophages and epithelial cells infected with different Chlamydia species and strains (Coutinho-Silva et al., 2001b, 2003b; Darville et al., 2007) by overriding Chlamydiae's evasion mechanisms. P2X7 receptor activation in Chlamydia-infected macrophages induces phospholipase D activation, intracellular Ca^+2^ mobilization, and subsequent phagolysosome formation and acidification (Coutinho-Silva et al., 2003b). P2X7 genetic deletion increased inflammation in the endocervix, oviduct, and mesosalpingeal tissues in a model of Chlamydia vaginal infection, making P2X7 KO mice more susceptible to infection than wild-type mice (Darville et al., 2007). In addition, P2X7 receptor is important for NLRP3 inflammasome activation and IL-1β secretion, which contributes to an efficient immune response against Chlamydiae infections (Abdul-Sater et al., 2010; He et al., 2010; Shimada et al., 2011; Nagarajan et al., 2012). Therefore, these reports suggest a role for P2X7 receptor in the control of Chlamydiae infections by increasing the microbicidal mechanisms in infected cells and the immune response against these bacteria.

### *Porphyromonas gingivalis* infection

The importance of P2X7 receptor in the immune response against bacteria involved in the pathogenesis of periodontitis, such as *Porphyromonas gingivali*s, has been explored over the last decade (reviewed in Almeida-da-Silva et al., 2016). *P. gingivali*s is an intracellular bacteria member of the polymicrobial dental biofilm community, which is involved in the pathogenesis of periodontitis. *P. gingivali*s infection induces ATP secretion from macrophage-derived THP-1 cells. Released ATP, in turn, activates P2X7 receptor, which is crucial for K^+^ efflux and NLRP3 inflammasome activation, inducing IL-1β secretion and pyroptotic cell death (Park et al., 2014). P2X7/P2X4 receptors are also required for ROS-mediated NLRP3 inflammasome formation and IL-1β secretion in *P. gingivalis*-infected gingival epithelial cells (Choi et al., 2013; Hung et al., 2013).

However, *P. gingivalis* has an ATP-consuming enzyme called nucleoside diphosphate kinase (NDK), a strategy to subvert these microbicidal mechanisms triggered by P2X7 receptor activation. When secreted, NDK metabolizes eATP, inhibiting ATP/P2X7 receptor-mediated ROS production and apoptosis of gingival epithelial cells (Yilmaz et al., 2008; Choi et al., 2013). Moreover, *P. gingivalis* fimbriae impair P2X7-dependent IL-1β secretion in murine macrophages (Morandini et al., 2014a).

Finally, Ramos-Junior et al. (2015) showed the importance of this receptor for the adaptive immune response during *P. gingivalis* infection by using P2X7 receptor deficient mice. Draining lymph node cells from P2X7 KO mice produced less IL-17 and IFN-γ compared to cells from WT-infected mice (Ramos-Junior et al., 2015). Therefore, P2X7 receptor seems to be a potential target for the development of new strategies to treat periodontitis. Additional detailed information regarding the role of P2X7 receptor and inflammasomes in *P. gingivalis* can be found in a recent review (Almeida-da-Silva et al., 2016).

### Mycobacterial infections

P2X7 receptor expression increases in peripheral blood mononuclear cells from patients with tuberculosis (Franco-Martínez et al., 2006). Also, P2X7 receptor's loss-of-function polymorphisms have been linked to an increased susceptibility to pulmonary (Sharma et al., 2010; Areeshi et al., 2015; Wu et al., 2015; Shamsi et al., 2016) and extra-pulmonary tuberculosis (Fernando et al., 2007; Sharma et al., 2010; Ben-Selma et al., 2011; Singla et al., 2012). This elevated susceptibility was correlated with an impaired capacity of macrophages to eliminate the bacillus (Fernando et al., 2007).

Molloy et al. (1994) showed for the first time that eATP is able to induce mycobacterial killing in infected human monocytes. Several subsequent studies described that eATP, acting via P2X7 receptor, induces mycobacterial killing in infected macrophages by mechanisms which are dependent on phospholipase D activation and apoptosis of infected cells (Kusner and Adams, 2000; Fairbairn et al., 2001; Placido et al., 2006). Interestingly, *in vivo* studies have shown that the susceptibility of P2X7 KO mice to mycobacterium infection depends on the strains, virulence and severity of the infection. Santos et al. (2013) reported that P2X7 deficient mice were more susceptible to mycobacterium infection when exposed to a high dose of the common laboratory strain H37Rv. On the other hand, P2X7 KO mice infected with hypervirulent strains (Beijing1471 or MP287/03) were less susceptible to infection than WT mice (Amaral et al., 2014).

The reduced susceptibility of P2X7 KO mice is verified because hypervirulent mycobacteria lead to macrophage death by necrosis in a P2X7 receptor-dependent manner increasing the bacillus release. So, the deleterious effects of P2X7 receptor observed in hypervirulent mycobacteria infection may be related to a vicious cycle where high eATP amounts are released by damaged cells, exacerbating inflammation, lung damage, and bacillus spreading (Amaral et al., 2014). Bomfim et al. (2017) recently showed that bone marrow-derived cells expressing P2X7 receptor have a critical role in promoting progression of severe tuberculosis. Therefore, P2X7 receptor is a key player in the modulation of immune responses against mycobacterium infections presenting protective or deleterious effects depending on mycobacterial strains.

### Bacterial sepsis

Sepsis is the leading cause of death in intensive care units worldwide and represents a major public health issue. During this life-threatening condition, the body's unfettered inflammatory response to a pathogen, commonly bacteria, triggers a cascade of events, which culminate in death by multiple organ failure (Singer, 2016). Plasma ATP levels increase in sepsis, suggesting a possible role for this pro-inflammatory molecule in the development of excessive systemic inflammation, characteristic of the disease (Cauwels et al., 2014; Sumi et al., 2014). The potential role of ATP and purinergic signaling in the pathophysiology of sepsis has been explored in humans and different animal models over the last few years (Haskó et al., 2011; Vuaden et al., 2011; Cauwels et al., 2014; Sumi et al., 2014; Csóka et al., 2015a,b; Ledderose et al., 2016; Savio et al., 2017a).

Two putative genotypes composed of five functional P2X7 receptor single nucleotide polymorphisms (SNPs) have been identified in patients with sepsis (Geistlinger et al., 2012). In the sequence, P2X7 functionality tests in dendritic cells isolated from healthy individuals that carry the same SNPs previously identified in septic patients were performed. These tests revealed an improved P2X7 receptor functionality, characterizing those as gain-of-function SNPs with deleterious effects in sepsis (Geistlinger et al., 2012). Animal models support this assumption by demonstrating that P2X7 receptor contributes to the production of inflammatory cytokines and exacerbates inflammatory response in sepsis (Santana et al., 2015; Wang et al., 2015a; Yang et al., 2015; Savio et al., 2017a,b; Wu et al., 2017a). P2X7 receptor genetic deletion improved survival rate by 30% as compared to septic WT mice (Santana et al., 2015). Similarly, Wang et al. (2015a) reported that A438079-treated WT mice or P2X7 KO mice showed a significant reduction in the mortality rate (50%) in a sepsis model of cecal ligation puncture (CLP) surgery.

Moreover, systemic administration of BzATP increases inflammation, while the systemic blockade of P2X7 receptor with A740003 decreases levels of serum and intestinal mucosal pro-inflammatory cytokines after CLP (Wu et al., 2017a). The reduced inflammatory response in A740003-treated mice protects them against sepsis-induced intestinal barrier disruption (Wu et al., 2017a). BBG treatment has also been shown to improve outcomes in sepsis. We recently showed that BBG improves survival, reduces cytokine production, activation of inflammatory signaling pathways, and liver injury in CLP-induced sepsis (Savio et al., 2017a). Accordingly, BBG-treated mice showed significantly lower production of inflammatory mediators, such as IL-1β and TNF-α, as well as a tendency toward better survival rate after intravenously administration of a uropathogenic α-haemolysin producing *E. coli* strain (ARD6) (Greve et al., 2017).

Other studies suggest that P2X7 receptor activation might be protective in sepsis by enhancing macrophage's ability to kill bacteria, and increasing secretion of IgM by B1 cells lymphocytes (Proietti et al., 2014; Csóka et al., 2015a). Intriguingly, Csóka et al. (Csóka et al., 2015a) showed higher levels of inflammatory chemokines and cytokines and increased mortality rate in P2X7 KO mice after sepsis by CLP compared to WT mice. Csóka et al. (2015a) also demonstrated increased cytokine levels in septic mice treated with oATP (an irreversible non-specific P2X7 antagonist), conflicting with several reports conferring anti-inflammatory properties to oATP by inhibition of P2X7 receptor, and other inflammatory signaling pathways (i.e., MyD88/NF-kB) and cytokine production *in vitro* and in models of autoimmune disease (Beigi et al., 2003; Vergani et al., 2013a,b; Savio et al., 2017a).

As in Csòka's findings, Greve et al. (2017) also showed increased susceptibility of P2X7 KO mice from GlaxoSmithKline (Brentford, UK)—mice that express a functional P2X7 protein in T cells—to a severe model of sepsis, in which mice received a high dose (165 million) of ARD6 *E. coli* strain intravenously. However, no significant differences were observed in bacterial load and plasma TNF-α, keratinocyte chemoattractant (KC), IL-1β and IL-6 between P2X7 KO and WT mice, 2.5 h after injection of a high dose of ARD6. Surprisingly, increased levels of IL-1β and IL-6, but not KC and TNF-α were reported in P2X7 KO mice injected with a low dose (41 million) of ARD6. The authors propose that the higher production of IL-1β in P2X7 KO receptor mice occurs via a non-canonical caspase8-dependent mechanism (Greve et al., 2017).

Finally, opposing results regarding the P2X7 receptor in sepsis could be related to differences in the mice microbiome, bacterial virulence, and severity of the infection. Although the P2X7 receptor may be relevant to bacterial control, morbidity, and mortality in this complex disease are related to onset of shock and hemodynamic compromise with multiple organ dysfunction and failure due to the excessive, unfettered inflammation in response to a pathogen. Since studies in humans and animal models have shown that P2X7 receptor contributes to the exacerbated inflammatory response, the use of P2X7 receptor inhibitors in addition to the antibiotic therapy seems to be a promising therapeutic strategy for sepsis management.

### P2X7 receptor in fungal infections

A limited number of studies have explored the role of P2X7 receptor in fungal infections. Perez-Flores et al. (2016) reported that P2X7 receptor in J774 cells functions as a scavenger receptor for bacteria but not yeast. P2X7 receptor is not required for NLRP3 activation and IL-1β secretion during *Candida albicans* infection, an opportunistic fungal pathogen. *In vitro*, P2X7 KO and WT macrophages secreted similar levels of IL-1β in response to *C. albicans* infection (Hise et al., 2009).

On the other hand, Xu et al. (2016) showed that invariant natural killer T (iNKT) cells interact with DC and release ATP, which activates P2X7 receptor in DC. P2X7 receptor activation in DC induces secretion of prostaglandin E2 (PGE2) stimulating neutrophil recruitment and controlling *C. albicans* infection. P2X7 deficient mice were more susceptible to pulmonary paracoccidioidomycosis, a disease caused by the dimorphic fungus *Paracoccidioides brasiliensis* (Feriotti et al., 2017). P2X7 receptor is crucial for the induction of an efficient Th1/Th17 immune response against *P. brasiliensis* in an NLRP3-dependent manner (Feriotti et al., 2017). Although P2X7 receptor may contribute to immune responses against certain fungal infections, further studies evaluating the relevance of pharmacological manipulation of P2X7 receptor in this field are necessary.

### P2X7 receptor in protozoa infections

Over the last decade, our laboratory has been exploring the role of P2X7 receptor in Leishmaniasis, a neglected tropical disease caused by a parasite of the genus Leishmania (Alvar et al., 2012). P2X7 receptor expression is upregulated in *Leishmania amazonensis-*infected macrophages and its activation reduces parasitic load in these cells (Chaves et al., 2009). Mechanistically, we demonstrated that activation of P2X7 receptor in infected macrophages reduces parasitic load via leukotriene B4 (LTB4) production (Chaves et al., 2009, 2014). Moreover, macrophage infection with *L. amazonensis* induced P2X7 receptor-mediated membrane permeabilization to anionic dye molecules, while it strongly decreased the uptake of cationic dyes (Marques-da-Silva et al., 2011). Interestingly, oATP affects parasite integrity and macrophage function by inhibiting the attachment/entrance of *L. amazonensis* promastigotes in a P2X7 receptor-independent manner (Figliuolo et al., 2014).

Recently, we showed that P2X7 receptor modulates the balance between inflammation and parasite control through a series of *in vivo* experiments where we infect P2X7 KO and WT mice with *L. amazonensis*. In a mouse model where subcutaneous *L. amazonensis* infection is induced by subcutaneous injection of the parasite in the footpad, genetic deletion of P2X7 receptor resulted in increased cell infiltration, higher IFN-γ levels, and low concentrations of IL-17 and TGF-β in the footpad, suggesting an excessive pro-inflammatory response. In addition to that, CD4^+^ and CD8^+^ T cells from infected P2X7 KO mice exhibited higher proliferative capacity than cells from infected WT mice (Figliuolo et al., 2017a). P2X7 KO mice were more susceptible to *L. amazonensis* infection showing increased lesion size and parasitic load (Figliuolo et al., 2017a). Increased susceptibility of P2X7 KO mice to *L. amazonensis* might be related to the role of P2X7 receptor in controlling T cell proliferation by inducing apoptosis. P2X7 KO mice showed an excessive CD4^+^ T cell proliferation which has a pathogenic role in leishmaniasis (Figliuolo et al., 2017a). Thus, P2X7 receptor is crucial for the immune response during leishmaniasis by regulating both innate and adaptive immunity.

P2X7 receptor also plays a key role in the microbicidal mechanisms that control infection by *Toxoplasma gondii*, a protozoan parasite that infects most species of warm-blooded animals causing toxoplasmosis. P2X7 receptor activation in *T. gondii* infected macrophages mediates pathogen elimination through ROS production and acidification of the parasitophorous vacuole (Corrêa et al., 2010; Lees et al., 2010). Mechanistically, we recently showed that P2X7 receptor controls *T. gondii* infection *via* ROS generated from NADPH oxidase, while IL-1β stimulates mitochondrial ROS production (Moreira-Souza et al., 2017). In intestinal epithelial cells, P2X7 receptor induces production of cytokines and chemokines that contribute to host immune response against *T. gondii* infection (Huang et al., 2017). P2X7 KO mice showed increased susceptibility to toxoplasmosis characterized by an impaired production of pro-inflammatory cytokines (IL-1β, IL-12, TNF-α, and IFN-γ) and increased tissue damage and parasitic load (Miller et al., 2011, 2015; Corrêa et al., 2017; Huang et al., 2017). Accordingly, loss-of-function SNPs in the human P2X7 receptor gene have been directly associated with increased susceptibility to toxoplasmosis by generating less functional proteins (Jamieson et al., 2010). Therefore, P2X7 receptor functionality is crucial to mount an efficient immune response against *T. gondii* infection.

P2X7 receptor relevance in the pathophysiology of Chagas disease or American trypanosomiasis, a tropical disease caused by the parasite *Trypanosoma cruzi*, has also been explored. By mechanisms still unknown, this parasite potentiates P2X7 receptor sensitivity to ATP, increasing plasma membrane permeabilization, calcium signaling, and death of CD4^+^/CD8^+^ double positive thymocytes during the atrophy phase of disease in a murine model (Mantuano-Barradas et al., 2003). Nevertheless, experiments performed with P2X7 KO mice suggested that this receptor might not be critically involved in *T. cruzi-*mediated thymus atrophy (Cascabulho et al., 2008). On the other hand, P2X7 KO mice showed an increased migration of mast cells toward the inflamed heart, which may compromise the innate immune response against *T. cruzi* (Meuser-Batista et al., 2011). Importantly, P2X7 receptor expression and functionality is not altered in peripheral lymphocytes from patients with Chagas Disease (Souza et al., 2017).

The role of P2X7 receptor in Malaria, a blood disease caused by the plasmodium parasite transmitted by the Anopheles mosquito, has also been explored since plasmodium-infected erythrocytes release large amounts of ATP (Akkaya et al., 2009). Extracellular ATP increases Ca^2+^ levels in *P. falciparum* modulating parasite invasion. Interestingly, treatment with pam P2 inhibitor suramin or apyrase before infection significantly reduces invasion of red blood cells by *P. falciparum* (Levano-Garcia et al., 2010). Recently, we have shown that ATP released by rupture of infected erythrocytes activates P2X7 receptor in CD4^+^ T inducing a Th1 immune response during blood-stage *Plasmodium chabaudi* malaria. P2X7 receptor deficient mice were more susceptible to plasmodium infection due to an impaired Th1 immune response (Salles et al., 2017). Therefore, P2X7 receptor is important for the development of an efficient immune response against plasmodium infection.

Finally, Mortimer et al. (2015) described that infection by *Entamoeba histolytica*, an extracellular human parasite that causes intestinal and extra-intestinal amebiasis, induces α_5_β_1_ integrin activation and ATP release through pannexin-1 channels activating P2X7 receptor and, consequently, NLRP3 inflammasome in macrophages. These results highlight the role of ATP-P2X7-NLRP3 inflammasome axis in the inflammatory immune response during amebiasis.

Overall, these data support a role for ATP-P2X7 receptor in boosting the immune system against the protozoa infections. Nevertheless, peculiarities of each parasite infection should be considered when developing pharmacological treatments targeting P2X7 receptor during protozoan infections.

### P2X7 receptor in helminth infections

Helminths are worm-like parasites that currently infect more than 1 billion people worldwide (Hotez et al., 2014). These worms usually induce a type 2 immune response, increasing susceptibility to infections by other lethal pathogen, being a serious socioeconomic problem in developing countries (Cortés et al., 2017).

P2X7 receptor has been implicated in the immune response against helminth parasites. Recently, Shimokawa et al. (2017) showed that P2X7 receptor activated by ATP released from intestinal epithelial cells induces the production of IL-33 by mast cells, which is crucial for induction of IL-13-producing group 2 innate lymphoid cells and clearance of helminth infections. In addition, soluble products derived from Trichuris *suis* (a type of helminth) downregulated P2X7 receptor expression in DC and macrophages. Co-stimulation of DC and macrophages with LPS and *T. suis* soluble products caused lower levels of IL-1β compared to LPS-primed cells, indicating a reduction in the activation state and inflammatory response of human macrophages (Ottow et al., 2014).

Purinergic signaling is also altered during Schistosomiasis, a chronic inflammatory disease caused by blood fluke worms belonging to the genus Schistosoma (Oliveira et al., 2013, 2014, 2016; Silva, 2016). P2X7 receptor functionality, as assessed by ATP-mediated Ca^2+^ influx and cell permeabilization, is reduced in mesenteric endothelial cells (Oliveira et al., 2013) and peritoneal macrophages from *S. mansoni* infected mice. This reduction is caused by increased TGF-β1 levels, which can reduce P2X7 receptor cell surface expression (Oliveira et al., 2014). Moreover, P2X7 KO mice infected with *S. mansoni* died 60 days after the infection, while no death was observed in *S. mansoni*-infected WT mice (Oliveira et al., 2014), suggesting that this receptor is crucial for an efficient immune response against this worm.

Taken together, these reports showed that helminth infections downregulate P2X7 functionality and expression. Since this receptor is crucial for controlling these infections, the use of P2X7 agonists could be a therapeutic strategy to boost the immune response against these worms.

## P2X7 receptor in inflammatory disease—a demon via exacerbation of the inflammatory state

P2X7 receptor is involved in several physiological and pathological conditions in different body systems by modulating cellular responses in immune and non-immune cells in a variety of disease (Burnstock, 2017b). In this section, we discuss the role of P2X7 receptor in inflammatory disease, including the respiratory tract, gut, liver, and renal diseases as well as in diabetes.

### P2X7 receptor in respiratory tract diseases

Purinergic signaling modulates important physiological processes in the airways. P2 purinergic receptors are expressed in different cells present in the respiratory system, such as type I alveolar epithelial cells, pulmonary endothelial cells, and resident immune cells (Mishra et al., 2011; Burnstock et al., 2012). Mishra et al. (2011) reported that P2X7 receptor activation with BzATP increases surfactant secretion in primary cultures of alveolar epithelial cells. However, this effect was blocked by BBG treatment. In addition, P2X7 KO mice showed impaired surfactant secretion in response to hyperventilation, highlighting the physiological importance of this receptor in lung homeostasis (Mishra et al., 2011).

However, P2X7 receptor hyperactivation has been implicated in the pathogenesis of a variety of respiratory diseases, including pulmonary hypertension, asthma, chronic obstructive pulmonary disease, acute lung injury, and pulmonary fibrosis (reviewed in Burnstock et al., 2012; Gentile et al., 2015). Increased ATP concentrations were detected in bronchoalveolar fluid of patients with idiopathic pulmonary fibrosis as well as in the bronchoalveolar space of mice subjected to a model of lung inflammation induced by LPS (Riteau et al., 2010) or cigarette smoking (Baxter et al., 2014).

Once released in the extracellular medium during lung injury, eATP acts as a danger signal through activation of P2X7 receptor, inducing alveolar macrophage activation and secretion of IL-1β (Riteau et al., 2010; Wang et al., 2015b; Galam et al., 2016) and IL-1α (Dagvadorj et al., 2015), and neutrophil recruitment (Riteau et al., 2010; Monção-Ribeiro et al., 2011; Baxter et al., 2014; Dagvadorj et al., 2015; Wang et al., 2015b; Mishra et al., 2016). The activation of P2X7 receptor increases markers of tissue fibrosis, such as lung collagen content and tissue inhibitor of matrix metalloproteinase-1 (TIMP-1) during lung injury (Riteau et al., 2010; Monção-Ribeiro et al., 2011). All these deleterious effects were attenuated in P2X7 KO mice (Riteau et al., 2010; Monção-Ribeiro et al., 2011; Baxter et al., 2014; Dagvadorj et al., 2015; Galam et al., 2016) or pharmacological inhibition of P2X7 receptor with A438079 (Wang et al., 2015b), BBG (Wang et al., 2015b), and suramin, a broad spectrum P2 receptor antagonist (Riteau et al., 2010).

Experiments performed *in vitro* co-cultures of human lung epithelial and macrophage cell lines suggest that P2X7 receptor also participates in lung inflammation triggered by nanoparticles such as SiO2 and TiO2, through the induction of inflammasome activation and IL-1β secretion (Dekali et al., 2013). Moreover, Monção-Ribeiro et al. (2014) showed that P2X7 receptor activation contributes to silica-induced lung fibrosis. P2X7 receptor deficient or BBG-treated mice showed diminished lung inflammation and fibrosis, as well as improved pulmonary function after silica instillation (Monção-Ribeiro et al., 2014). Therefore, P2X7 receptor contributes to lung inflammation and fibrosis, being an attractive therapeutic target to treat diseases of the respiratory tract.

### P2X7 receptor in gut disease

Several reports have described the participation of purinergic signaling in pathophysiological mechanism of gut diseases (reviewed in Burnstock, 2016; Longhi et al., 2017). P2X7 receptor expression and functionality have been characterized in different gut compartments (de Campos et al., 2012), and shown to be upregulated in colon of patients with Crohn's disease (CD) (Neves et al., 2014).

Rodent animal models of inflammatory bowel disease have shown that activation of P2X7 receptor induces death of enteric neurons and intestinal inflammation by activation of mast cells in the gut, leading to intestinal motility disorders (Gulbransen et al., 2012; Kurashima et al., 2012; Antonioli et al., 2014; Matsukawa et al., 2016). In this context, pharmacological inhibition of P2X7 receptor by BBG (Marques et al., 2014), A438079 (Wan et al., 2016), and A740003 (Marques et al., 2014; Figliuolo et al., 2017b) in animal models of inflammatory bowel disease showed beneficial effects attenuating the inflammatory response in the colon. Likewise, genetic deletion of P2X7 receptor protects mice against chemically induced colitis (Neves et al., 2014; Figliuolo et al., 2017b). Mechanistically, we recently demonstrated that eATP, released from damaged cells or pathogenic bacteria, contributes to chemically-induced colitis by triggering P2X7 receptor-mediated Treg cell death (Figliuolo et al., 2017b). P2X7 receptor deletion increases the expression of CD103, an integrin involved in T lymphocytes addressing the intestinal epithelium, in mesenteric lymph nodes of mice with 2,4,6-Trinitrobenzenesulfonic acid solution (TNBS)-induced colitis. In this model, Treg cells leave the mesenteric lymph nodes and migrate to lamina propria in greater numbers in P2X7 receptor KO than WT mice. Increased number of activated Treg cells, leading to higher levels of anti-inflammatory cytokines, such as IL 10 and TGF-β, in the lamina propria of P2X7 receptor KO mice protects them against experimental colitis (Figliuolo et al., 2017b).

In contrast, studies reported by Hashimoto-Hill et al. (2017), using a different model of colitis induced by *Citrobacter rodentium* infection, suggest a protective role for upregulation of P2X7 receptor expression in the gut. In their studies, retinoic acid-induced P2X7 receptor expression in CD4^+^ effector T cells in the intestine, by activation of the intragenic enhancer region of the P2X7 receptor mouse gene, inhibited excessive expansion of Th1 and Th17 cells in the intestine during colitis (Hashimoto-Hill et al., 2017).

Taken together, these reports support the involvement of P2X7 receptor in inflammatory mechanisms underlying inflammatory bowel diseases. Interestingly, a clinical trial evaluating the effects of a selective orally active inhibitor of the P2X7 receptor (AZD9056) showed beneficial effects in patients with moderate-to-severe Crohn's Disease (Eser et al., 2015). Therefore, this receptor seems to be a suitable therapeutic target to treat gut disease.

### P2X7 receptor in liver disease

Purinergic signaling has been implicated in liver inflammation (Vaughn et al., 2012). Particularly, the P2X7 receptor is involved in oxidative and inflammatory mechanisms, as well as fibrogenesis, by inducing activation of Kupffer cells and hepatic stellate cells in several liver diseases (Vaughn et al., 2012). P2X7 receptor blockade with A438079 attenuates mouse CCl_4_-induced liver fibrosis (Huang et al., 2014). BBG or oATP treatment also reduces the production of inflammatory mediators, activation of inflammatory signaling pathways and liver fibrosis in a model of bile duct ligation-induced cirrhosis (Tung et al., 2015).

Savio et al. (2017a) reported that P2X7 receptor blockade with BBG protects mice against sepsis-induced liver injury by decreasing activation of inflammatory signaling pathways, cytokine production, and apoptosis of sinusoidal cells. Pretreatment with BBG, followed by administration of celastrol, a pentacyclic triterpene isolated from plants with anti-inflammatory activity, also protects the liver from acetaminophen-induced liver injury by reducing inflammation and oxidative damage (Abdelaziz et al., 2017). In a model of carbon tetrachloride-induced steatohepatitis in obese mice, P2X7 receptor mediates oxidative and inflammatory events in the liver by NADPH oxidase-dependent mechanisms. P2X7 receptor is required for NADPH oxidase activation by inducing the expression of the p47 phox subunit and p47 phox binding to the membrane subunit, gp91 phox, suggesting that P2X7 receptor is involved in oxidative damage in liver disease (Chatterjee et al., 2012). According to Das et al. (2013) this receptor is also crucial for oxidative stress-mediated autophagy, inflammation, and fibrosis in liver during experimental non-alcoholic steatohepatitis in P2X7 KO mice (Das et al., 2013).

P2X7 receptor also contributes to acetaminophen hepatotoxicity in mice (Hoque et al., 2012) and to hepatic stellate cell activation and liver fibrogenesis via NLRP3 inflammasome and PKC/GSK3β pathways (Jiang et al., 2017; Wu et al., 2017b). P2X7 receptor stimulation by BzATP induced the expression of inflammatory mediators and proteins related to fibrosis development, such as collagen I, in hepatic stellate cells treated with acetaldehyde. These effects were attenuated by P2X7 receptor inhibition with A438079 (Wu et al., 2017b). Therefore, P2X7 receptor blockade might be an innovative therapeutic avenue to limit the activation of Kupffer and hepatic stellate cells, and, consequently, the inflammation and fibrogenesis in liver diseases.

### P2X7 receptor in diabetes

Purinergic signaling has been studied in pancreas physiology and pathophysiology since the 1960's, when Rodrigue-Candela et al. (1963) showed that ATP induces secretion of insulin in rabbit pancreas slices. Currently, the role of several purinergic receptors and ectonucleotidases in pancreas physiology and diabetes is widely accepted (Burnstock and Novak, 2012, 2013). Specifically, P2X7 receptor has been implicated in diabetes development and pathology (Coutinho-Silva et al., 2007; Burnstock and Novak, 2013). Polymorphisms in P2X7 receptor gene have been associated with altered glucose homeostasis in both mice and humans (Todd et al., 2015). In rat and mice, P2X7 receptor is expressed in α cells, but not in β or δ cells, an expression pattern that remains unchanged during aging or after streptozotocin (STZ) administration (Coutinho-Silva et al., 2001a, 2003a). Besides that, P2X7 receptor-mediated apoptosis is increased in fibroblasts from type 2 diabetes patients (Solini et al., 2004) and in human fibroblasts exposed to high glucose levels (Solini et al., 2000). In addition, osteoblast function is impaired in a P2X7-dependent manner after high glucose exposure (Seref-Ferlengez et al., 2016). Interestingly, Weitz et al. (2017) recently showed that high glucose levels also induce ATP secretion from β cells triggering the activation of pancreatic islet-resident macrophages.

Using P2X7 receptor deficient mice, Glas et al. (2009) reported that these mice subjected to a high-fat/high-sucrose diet have diminished insulin secretion and glucose tolerance correlated with an elevated number of apoptotic β cells. However, Chen et al. (2011) reported that P2X7 genetic deletion did not alter type 1 diabetes development in non-obese mice, while Vieira et al. (2016) showed that P2X7 KO mice are resistant to STZ-induced type 1 diabetes induction. No increase in blood glucose and a decrease in insulin-positive cells were reported in STZ-treated P2X7 KO mice in comparison to WT counterparts and an analysis of pancreatic lymph nodes showed a diminished inflammatory response in diabetic P2X7 KO mice. Finally, BBG treatment also prevented the STZ-induced diabetes (Vieira et al., 2016). Therefore, P2X7 receptor seems to be implicated in diabetes types 1 and 2.

P2X7 receptor also appears to be involved in diabetes-related comorbidities, including cardiovascular alterations, diabetic retinopathy, and kidney injury (Burnstock and Novak, 2013). P2X7 receptor expression is increased in renal biopsy sections from diabetic patients (Rodrigues et al., 2014; Menzies et al., 2017a) and in the kidney of STZ-induced diabetic rats contributing to diabetic nephropathy pathogenesis. P2X7 receptor blockade with AZ11657312 (Menzies et al., 2017a), aerobic training or N-acetylcysteine administration attenuated diabetes related kidney injury (Rodrigues et al., 2014). Therefore, these results suggest that P2X7 receptor is involved in the development of diabetes and associated comorbidities.

### P2X7 receptor in kidney disease

Purinergic signaling exerts crucial physiological effects on renal function, controlling both vascular contractility and tubular function (recently reviewed in Menzies et al., 2017b). eATP released by renal cells regulates renal homeostasis through activation of P2 purinergic receptors present in non-hematopoietic renal cells as well as infiltrating immune cells (Koo et al., 2017; Menzies et al., 2017b). Thus, an imbalance on purinergic receptors activation, as well as in ectonucleotidases activity, contributes to kidney diseases (Vuaden et al., 2012; Franco et al., 2015).

Uncontrolled activation of P2X receptors, mainly P2X7 receptor, has been shown to play a critical role in the inflammatory reactions observed in renal diseases, contributing to renal glomerular, tubular, and vascular damage (Gonçalves et al., 2006; Deplano et al., 2013; Solini et al., 2013; Franco et al., 2015). This assumption is supported by the fact that, in a healthy kidney, P2X7 receptor is weakly expressed in podocytes, endothelial cells, mesanglial cells, and tubular epithelial cells, while its levels become upregulated after injury (Gonçalves et al., 2006; Solini et al., 2013; Burnstock et al., 2014; Rodrigues et al., 2014; Franco et al., 2015).

In the same way, infiltrated inflammatory cells such as macrophages, DCs, and T cells increase their P2X7 receptor expression after renal damage (Ji et al., 2012a,b; Deplano et al., 2013; Koo et al., 2017). It is now clear that activation of P2X7 receptor promotes pathologic inflammation in several experimental models of renal diseases, such as glomerulonephritis (Deplano et al., 2013), diabetic nephropathy (Solini et al., 2013; Rodrigues et al., 2014), tubulointerstitial nephritis (Gonçalves et al., 2006), hypertensive nephropathy (Franco et al., 2015), acute kidney injury (Koo et al., 2017), and chronic kidney injury (Granata et al., 2015) via activation of NLPR3 inflammasome and consequent release of inflammatory cytokine, such as IL-1β and IL-18. Moreover, this receptor participates in the production of interstitial collagen, contributing to renal fibrosis (Gonçalves et al., 2006; Koo et al., 2017). Therefore, blocking P2X7 receptor activation may be a potential strategy to prevent or ameliorate renal damage. In fact, several studies have demonstrated a protective effect of P2X7 receptor antagonism (with BBG, oATP, AZ10606120, A-438079, and AZ11657312) or its genetic deletion in animal models of renal injury due, primarily, to anti-inflammatory actions (Gonçalves et al., 2006; Ji et al., 2012a,b; Deplano et al., 2013; Solini et al., 2013; Menzies et al., 2015; Yan et al., 2015; Koo et al., 2017).

In this sense, P2X7 receptor appears to be an attractive therapeutic target to reduce inflammation in renal diseases. Unfortunately, recent clinical trials failed to demonstrate a beneficial effect for P2X7 receptor antagonists in several inflammatory illnesses (Arulkumaran et al., 2011; Menzies et al., 2017b). Additional detailed information regarding P2X7 receptor role in renal disease and its potential use as a new target in renal disease therapy can be found in a recent review (Menzies et al., 2017b).

### P2X7 receptor in cardiovascular disease

Purinergic signaling and P2X7 receptor also play a key role in cardiovascular physiology and pathophysiology. P2X7 receptor shows deleterious effects in cardiovascular disease by promoting inflammation, thrombosis, and endothelial dysfunction (Burnstock, 2017a). Additional detailed information can be accessed in an excellent recent review by Burnstock (2017a).

## P2X7 receptor in cancer—angel or demon depending on its level of activation and cell type studied

Despite recent advances in research and care, cancer is still a major public health problem worldwide and it is the second leading cause of death in the United States (Siegel et al., 2017). Recent research has granted both pro- and anti-carcinogenic properties to eATP in tumor biology. The definitive effect of eATP will depend on its concentration in the tumor intersticium, the panel of P2 receptor subtypes expressed by the tumor core and the levels of the ectonucleotidades expressed by immune, tumor, and stromal cells (Di Virgilio, 2012). Increased metabolism of eATP by CD39 and CD73 sequential enzymatic activity generates adenosine in the tumor microenvironment (TME), which is a potent immunosuppressive mediator and promotes cancer cell growth (Ohta et al., 2006; Deaglio et al., 2007; Mello et al., 2017b).

Particularly, P2X7 receptor activation has been linked to conflicting effects on carcinogenesis, being beneficial in certain circumstances and injurious in others (Roger et al., 2015). In order to facilitate the understanding of P2X7 receptor role in tumor progression, we will divide this following sections according to its effect on tumor cells (pro-tumoral and anti-tumoral) and immune infiltrated cells, highlighting P2X7 receptor impact on the tumor-host interaction and its inhibition/activation as a new therapeutic target potential in cancer.

### P2X7 receptor basal stimulation in tumor cells—pro-tumoral activities

Growing body of evidence indicates that P2X7 receptor plays a central role in tumorigenesis (Adinolfi et al., 2009, 2012; Tafani et al., 2011; Amoroso et al., 2015). P2X7 inhibition, either by silencing or pharmacological blockade, restrained tumor progression, and metastasis while P2X7 overexpression accelerated it (Jelassi et al., 2011; Adinolfi et al., 2012). Moreover, high expression levels of P2X7 receptor have been found in many malignant human tumors, including leukemia (Adinolfi et al., 2002; Zhang et al., 2004; Chong et al., 2010), melanoma (Deli et al., 2007), neuroblastoma (Raffaghello et al., 2006), pancreatic adenocarcinoma (Giannuzzo et al., 2015), esophageal carcinoma (Santos et al., 2017), papillary thyroid carcinoma (Kwon et al., 2014), renal cell carcinoma (Liu et al., 2015), breast cancer (Tan et al., 2015), prostate cancer (Ghalali et al., 2014), colorectal cancer (Qian et al., 2017), and head and neck cancer (Bae et al., 2017).

It is now consensus that under conditions of low-intensity basal stimulation, P2X7 receptor has a potent pro-growth activity, contributing to tumor progression (Di Virgilio, 2012). P2X7 stimulation results in increased oxidative phosphorylation and aerobic glycolysis, both events leading to an overall enhancement in the total cellular ATP content (Adinolfi et al., 2005; Amoroso et al., 2012) and consequent proliferative advantage (Di Virgilio, 2016). P2X7 receptor activates several intracellular growth promoting pathways, such as NFATc1, ERK, PI3K/Akt, and HIF-1α (Jacques-Silva et al., 2004; Tafani et al., 2011; Adinolfi et al., 2012; Amoroso et al., 2012, 2015). It also triggers the release of vascular endothelial growth factor (VEGF) (Hill et al., 2010; Adinolfi et al., 2012), tissue factor and matrix metalloproteinases (Gu and Wiley, 2006), contributing to tumor growth, angiogenesis, invasiveness, and metastasis spreading. P2X7 receptor role in tumor metastasis is also supported by the fact that its silencing leads to reduced expression of genes linked to epithelial/mesenchymal transition such as Snail, E-cadherin, Claudin-1, IL-8, and MMP-3 (Qiu et al., 2014).

Generally, the P2X7 receptor exhibits striking oncogene-like features, being therefore a potential target in cancer therapy. Many studies using P2X7 inhibitors and antagonist have rendered promising results, reducing cancer growth or spreading in preclinical animal models of colon cancer (Adinolfi et al., 2012), breast cancer (Jelassi et al., 2011; Park et al., 2016), ovarian cancer (Vázquez-Cuevas et al., 2014), pancreatic ductal adenocarcinoma (Giannuzzo et al., 2016), neuroblastoma (Amoroso et al., 2015), melanoma (Adinolfi et al., 2012, 2015), glioma (Ryu et al., 2011), and mesothelioma (Amoroso et al., 2016). The pharmacological molecules used to block P2X7 in those studies were administrated either intratumorally or systemically and included oATP (Adinolfi et al., 2012; Hattori and Gouaux, 2012), BBG (Vázquez-Cuevas et al., 2014), AZ10606120 (Adinolfi et al., 2012, 2015; Amoroso et al., 2015, 2016; Giannuzzo et al., 2016), A740003 (Adinolfi et al., 2015; Amoroso et al., 2015), A438079 (Jelassi et al., 2011), Antraquinone emodin (Jelassi et al., 2013), novel 1-Piperidinylimidazole based antagonists (Park et al., 2016), and P2X7 blocking antibodies (Ren et al., 2010). Many pharmaceutical companies are attempting to develop a clinical candidate targeting P2X7 receptor and various scaffolds have been disclosed (Park and Kim, 2017). A recent clinical trial in phase I, which used an anti-P2X7 antibody, provided significant data to support its use as a safe and tolerable topical therapy for basal cell carcinoma (BCC) (Gilbert et al., 2017). According to this study, 65% of patients responded to the treatment, having a significant reduction in the lesion area.

Considering P2X7 receptor role in promoting tumor angiogenesis via increased HIF1α activity and VEGF secretion (Adinolfi et al., 2012; Amoroso et al., 2012, 2015), it is tempting to say that the use of antagonists of P2X7 in association with anti-angiogenic drugs would surge synergistic effects in controlling tumorigenesis. In fact, administration of the VEGF antagonist Avastin strongly inhibits growth of P2X7-expressing tumors *in vivo* (Adinolfi et al., 2012).

Although outcomes of pre-clinical and clinical studies are exciting, novel P2X7-targeted strategy would only be feasible in tumors that overexpress this receptor, as tumors with low or no P2X7 expression showed slight response to P2X7 blocking therapy in preclinical models (Adinolfi et al., 2012).

### P2X7 receptor over-activation in tumor cells—anti-tumoral activities

Overstimulation of P2X7 receptor with high levels of exogenous ATP in order to produce tumor cell death is a longstanding observation and it was demonstrated in many types of cancer, such as colon cancer (Bian et al., 2013; Mello et al., 2017a), prostate cancer (Shabbir et al., 2008), cervical cancer (Wang et al., 2004; Mello et al., 2014), endometrial cancer (Li et al., 2006), melanoma (White et al., 2009; Feng et al., 2011; Bian et al., 2013), squamous cell carcinomas (Deli and Csernoch, 2008), and glioma (Tamajusuku et al., 2010; Gehring et al., 2012, 2015), among others. However, despite its promising results in preclinical models, clinical trials have failed to demonstrate a potent anti-cancer effect for eATP administration in patients, with an improvement of the quality of life being the only positive effect observed (Agteresch et al., 2003; Beijer et al., 2009, 2010).

This lack of effect in patients might be explained by the fact that both tumor and other cells in the TME are resistant to the high ATP concentrations usually present in this inflammatory setting (Bianchi et al., 2014). The mechanism enrolled in such refractoriness to ATP stimulation has not been completely understood yet, but it appears to resultfrom P2X7 receptor uncoupling from intracellular death pathways, at least in some types of cancer (Raffaghello et al., 2006). Therefore, finding strategies to enhance eATP-P2X7 signaling in the TME by boosting P2X7 functional responses might be a better approach to overcome cancer cell death escape in those cases where P2X7 receptor is less responsive.

We have recently demonstrated that hyperthermia is a suitable strategy to augment ATP-elicited P2X7 functionality, thereby maximizing tumor cell death (Mello et al., 2017a). Hyperthermia alters plasma membrane fluidity facilitating P2X7 receptor pore opening and dilatation upon ATP stimulation resulting in increased colon cancer cell death. Moreover, when combined with cisplatin or mitomycin C, hyperthermia, and eATP potentiate chemotherapy cytotoxicity, enhancing the therapeutic efficacy of conventional cancer treatments. Likewise hyperthermia, shock wave and photodynamic therapy alter cells membrane permeability via ATP-P2X7 signaling, boosting hydrophilic drugs intake, and cell death induction (Pacheco et al., 2016; Qi et al., 2016). P2X7 receptor activation also modulates radiotherapy-killing activity (Gehring et al., 2012, 2015).

According to Gering et al. eATP-P2X7 signaling acts synergistically with radiotherapy potentiating glioma cell death (Gehring et al., 2012, 2015). Moreover, silencing of P2X7 receptor in animal model of glioblastoma prevented response to radiation, reinforcing that functional P2X7 receptor expression is essential for an efficient radiotherapy response in gliomas *in vivo* (Gehring et al., 2015). These studies combined bring a different approach in relation to the use of eATP-P2X7 signaling intervention, specifically regarding its property to increase plasma membrane permeability to boost the efficacy of other anti-cancer modalities. Importantly, this strategy would only be applicable to tumor cells expressing the P2X7(A) splice variant, which is the well-characterized full-length receptor capable of inducing pore formation and apoptosis (Rassendren et al., 1997). So far, both functional and non-functional splice variants have been described for the human P2X7 receptor (Cheewatrakoolpong et al., 2005; Feng et al., 2006a,b). The truncated P2X7(B) variant is a functional ion channel with a dominant positive effect, however it lacks the ability to form the large non-selective conductance pore (Giuliani et al., 2014). Differently, the non-functional splice variant P2X7(J) exerts a dominant negative effect (Feng et al., 2006a,b). In these settings, tumor cells predominantly expressing truncated/defective P2X7 receptor variant might fail to undergo cell pore formation and death even with agonist stimulus (Cheewatrakoolpong et al., 2005; Skarratt et al., 2005; Feng et al., 2006a,b).

### P2X7 receptor in tumor-associated immune cells

The TME is composed of different subsets of immune cells that interact with tumor cells to enable tumor growth and progression (de Visser et al., 2006; Hanahan and Weinberg, 2011). In this inflammatory microenvironment, accumulated eATP activates P2X7 receptor highly expressed in monocytes, macrophages, DCs, lymphocytes, and myeloid-derived suppressor cells (MDSCs), modulating the immune response against tumors (Fernando et al., 1999; Gudipaty et al., 2003; Idzko et al., 2014). In this scenario, P2X7 receptor activation on DCs is crucial for the anti-immune response (Ghiringhelli et al., 2009; Aymeric et al., 2010). Engagement of P2X7 receptor triggers NLRP3 inflammasome activation and IL-1β release with consequent stimulation of CD8^+^ and CD4^+^ mediated anti-tumor responses. This signaling is particularly important for the induction of immunogenic cell death after radiotherapy and some chemotherapeutic agents, dictating the efficacy of those treatments (Zitvogel et al., 2008; Ghiringhelli et al., 2009; Aymeric et al., 2010). Moreover, two studies showed that expression of P2X7 receptor by host immune cells has a fundamental role in restraining tumor growth and metastatic spreading (Adinolfi et al., 2015; Hofman et al., 2015). Accordingly, tumors implanted in P2X7 KO mice grow faster, lack inflammatory infiltrate—such as neutrophils, lymphocytes, and macrophages—and metastasize more readily. Furthermore, plasma and intratumoral levels of IL-1β and VEGF were significantly lower in P2X7 KO compared to WT strains (Adinolfi et al., 2015).

P2X7 receptor appears to hinder tumor growth by promoting DC/tumor cell interaction, cytokine release, chemotaxis, and infiltration of immune cells in the TME (Adinolfi et al., 2015; Di Virgilio, 2016). It also stimulates the release of immunosuppressive factors from MDSCs (Bianchi et al., 2014), and modulates macrophages to the M2-immunosuppressive phenotype, preventing tumor cell attack by natural killer and T cells (Noy and Pollard, 2014; Bergamin et al., 2015). In this complex scenario, the prevalence of one of those conflicting inflammatory responses will dictate whether a tumor will grow and metastasize or will be successfully hindered by the immune system (Hanahan and Weinberg, 2011; Di Virgilio, 2016). In fact, the observation that systemic administration of pharmacologic P2X7 receptor antagonist strongly inhibits tumor growth in immune-competent mice (Adinolfi et al., 2012, 2015), suggests that blocking P2X7 receptor is more beneficial for cancer regression. Evidence supporting this assumption comes from the fact that, in tumors overexpressing P2X7 receptor, such as neuroblastoma, the anti-tumoral effect of P2X7 antagonists is more pronounced in immune-competent than in immune-compromised (nude/nude) mice, indicating an additional contribution of the immune response against tumor in the former case (Amoroso et al., 2015; Di Virgilio and Adinolfi, 2017). This effect could be partially attributed to blockade of P2X7 receptor expressed by suppressor immune cells (i.e., MDSCs and M2-macrophages), restoring the anti-tumor immune response.

Therefore, targeting eATP/adenosine signaling is a promising strategy to increase anti-tumor immunity in the TME and many researchers have pointed the blockage of this signaling as the next generation of cancer immunotherapy (Allard et al., 2017; Mello et al., 2017b). Considering the diversity of cells, the complexity of signaling, and the contradictory effect of P2X7 receptor in the TME, the application of P2X7 receptor-blocking therapy to restrain tumor growth and boost the anti-tumor immunity will only be feasible after our complete understanding of its role in the host-tumor interaction.

## P2X7 receptor activation in neurodegenerative disorders—a demon via exacerbation of neuroinflammation

In the brain, extracellular nucleotides, mainly ATP, act as signaling molecules mediating neuron-neuron and neuron-glia communication through activation of ionotropic (P2X) and metabotropic (P2Y) purinergic receptors (Cisneros-Mejorado et al., 2015). P2X7 receptor is widely distributed in the brain (Yu et al., 2008) and it is abundantly expressed in microglia and neurons, as well as in other glial cells such as oligodendrocytes and astrocytes (Yu et al., 2008). P2X7 receptor plays a key physiologic role on neural axonal growth and modulation of neurotransmitter release from presynaptic terminals, where it is prominently present (Sperlágh et al., 2002; Alloisio et al., 2008). Its brief stimulation also induces non-cytotoxic glial activation. However, in pathologic situations, even mild perturbations, ATP can be released in large amounts from neurons and activated glial cells, as well as from dying cells at the site of injury, dramatically increasing eATP levels. Repeated and prolonged activation of P2X7 receptor by high levels of eATP induces formation of large non-selective membrane pores, in addition to the typical Ca^+2^ influx and K^+^ efflux through non-selective cationic channels, initiating a cascade of pro-inflammatory and pro-apoptotic events that culminate in cell death. In addition, P2X7 receptor pores may be a route of glutamate and ATP efflux from glia and neurons, further fueling inflammation and cytotoxicity (Di Virgilio et al., 2001; Liang and Schwiebert, 2005).

Neuroinflammation and gliosis are the most prominent aspects in the pathophysiology of many neurodegenerative diseases. Increasing evidence suggests that deregulated expression and activation of P2X7 receptor is an underlying key mechanism in the pathogenesis and progression of many pathological states of central nervous system (CNS), particularly due to its regulatory role in glial activation, integrity, and survival. Activation of P2X7 receptor signaling in microglia induces inflammasome formation, releases pro-inflammatory cytokines (IL-1β, IL-18, and TNFα) and reactive oxygen species such asROS, particularly superoxide (Parvathenani et al., 2003), which induces activation of NFκB signaling, upregulation of pro-inflammatory, and pro-apoptotic genes. This willresult in death of surrounding cells, including neurons (Parvathenani et al., 2003). ATP released from dying cells perpetuates the inflammatory and degenerative cycle.

This cascade of events has been proposed as a common pathological mechanism to many CNS diseases where an initial perturbation, either mechanical, inflammatory, cytotoxic or infectious, induces increased in eATP, triggering P2X7 receptor-dependent inflammatory and pro-apoptotic cascade. If that holds true, such deleterious effects commonly present in neurodegenerative diseases could potentially be manageable by antagonists of P2X7 receptor, irrespective of the primary cause.

In fact, extensive body of work has demonstrated the involvement of P2X7 receptor activation not only in microglia but also in neurons, oligodendrocytes, and astrocytes, in the development of multiple CNS disorders including Traumatic Brain Injury, Parkinson's disease, Alzheimer's disease, ischemia, epilepsy, Huntington's disease, and Multiple Sclerosis. In the next sections, we will focus on the role of P2X7 receptor in the development and possible treatment of the neurodegenerative disorders that most heavily affect patients worldwide: Alzheimer's disease (AD), Parkinson's disease (PD), Huntington's disease (HD), and Multiple Sclerosis (MS). A detailed coverage of the physiologic role of P2X7 receptor in the brain, as well as its role in other CNS disorders, is available in recent reviews (Sperlágh and Illes, 2014; Tewari and Seth, 2015; De Marchi et al., 2016).

### Role of P2X7 receptor in Alzheimer's disease (AD)

According to the latest World Alzheimer Report from the Alzheimer's Disease (AD) International (an international federation of Alzheimer associations around the world), 46 million people are living with AD or a related dementia, being more than 10 million people only in the United States (“World Alzheimer Report 2016 | Alzheimer's Disease International” 2017). AD is a progressive form of dementia characterized by increasing neuronal loss, mainly in cortex and hippocampus. Neuropathological hallmarks of this disease are the presence of neurofibrillary tangles, formed by the accumulation of intracellular inclusions of hyperphosphorylated tau protein, and the deposition of extracellular plaques of amyloid-β peptides (Aβ) (Koffie et al., 2012; Pooler et al., 2014).

AD pathogenesis is complex and it has not been completely understood yet. Aβ production and aggregation is still considered to be at the origin of AD pathology, while Aβ-induced microglia activation is believed to contribute to disease progression through secretion of pro-inflammatory mediators and cytotoxic agents (Hardy and Selkoe, 2002). Protein post-translation modifications that promote inflammation, specifically the ureido degenerative protein modification arginine citrullination and lysine carbamylation, have been also associated with initiation and progression of dementia and AD. Interestingly, Gallart-Palau et al. (2017) have demonstrated presence of P2X7 receptor modified by a dementia-specific citrulline residue in soluble brain proteome of AD. However, the contribution of this post-translational modification to P2X7 receptor's activity remains to be demonstrated. Upregulation of P2X7 receptor was observed in the brain of AD patients, as well as in the hippocampus of animal models of AD such as Tg2576 transgenic mice and rats following intrahippocampal Aβ injection. In both cases, P2X7 receptor brain expression was concentrated in areas of high Aβ plaques density and co-localized with activated microglia, identifying microglial P2X7 receptor as another player in the pathogenesis of AD (Parvathenani et al., 2003; McLarnon et al., 2006). More recently, *in vitro* autoradiography quantification of brain slices incubated with a potent iodinated radiotracer for P2X7 receptor, [^123^I]TZ6019, demonstrated higher binding in brains from P301C tau transgenic mice, a tauopathy mouse model of AD, compared to control (Jin et al., 2017). While the cause of increased P2X7 receptor in AD is still unknown, studies performed in human fetal microglia demonstrated a significant two-fold upregulation in P2X7 receptor expression following *in vitro* treatment with Aβ, suggesting a direct effect of this peptide on P2X7 receptor expression (McLarnon et al., 2006).

Aβ also induces ATP release from microglia (Kim et al., 2007). Released ATP, acting in an autocrine/paracrine manner, further augments microglial activation, and likely contributes to neuronal death (Rampe et al., 2004; Kim et al., 2007; Sanz et al., 2009). Furthermore, due to its potent chemoattractant properties, higher eATP levels contribute to the high density of activated microglia surrounding Aβ plaques, a common finding in the brain of AD patients. Most interestingly, the inability of Aβ to activate P2X7 receptor deficient microglia suggests a fundamental role for this receptor in microglial activation induced by Aβ (Sanz et al., 2009). Supporting this assumption, P2X7 receptor blockade with antagonists or P2X7 receptor silencing prevented Aβ-induced pro-inflammatory signaling in microglia (McLarnon et al., 2006; Ryu and McLarnon, 2008). Silencing of P2X7 receptor also enhanced Aβ phagocytosis by microglia, an important mechanism for Aβ clearance (Ni et al., 2013).

A possible involvement for P2X7 receptor in modulating amyloid precursor protein (APP) processing and Aβ peptides production has also been proposed (Delarasse et al., 2011; León-Otegui et al., 2011; Miras-Portugal et al., 2015). APP, a transmembrane protein present in neurons and glia, can be processed by α- and β-secretases, followed by sequential cleavage by γ secretases to produce soluble APPα (sAPPα) and amyloidβ (Aβ) fragments, respectively. While both pathways of APP processing occur in the CNS, in a healthy brain, APP is preferentially processed by α-secretases (non-amyloidogenic process), precluding the formation of Aβ and resulting in sAPPα accumulation, which has been shown to have neurotrophic and neuroprotective properties. However, in AD brain, for reasons still unknown, APP cleavage by β-secretases is more pronounced, resulting in accumulation of Aβ peptides in senile plaques (Murphy and LeVine, 2010). While the protective role of activation of P2Y2 receptor in inducing the non-amyloidogenic possessing of APP is well-established (Camden et al., 2005; Kong et al., 2009), the contribution of P2X7 receptor is debatable.

Delarasse et al. (2011) performed studies using human and mouse neuroblastoma cell lines that constitutively express APP and a functional P2X7 receptor. They showed that these cells release APP fragments when stimulated with both ATP and high levels of BzATP. Interestingly, APP fragments were secreted mostly as sAPPα while Aβ was undetectable, suggesting that P2X7 receptor activation promotes APP cleavage predominantly via α-secretase pathway. Moreover, this effect was abolished by both P2X7 receptor siRNA and pre-treatment with P2X7 receptor pharmacologic inhibitors, further validating the involvement of P2X7 receptor signaling in promoting sAPPα release. These results were also replicated in mouse primary astrocytes and neural progenitor cells, as well as in human neuroblastoma cells. In these cells, ATP increased sAPPα secretion in wild type cells, but failed to do so in P2X7 receptor knockout cells. This non-amyloidogenic APP cleavage induced by P2X7 receptor activation was mediated by metalloproteases, via activation of an ERK1/2, P38, and JNK signaling pathway, independently from typical P2X7 receptor induced Ca^+2^ influx (Delarasse et al., 2011). Such results suggest a protective effect of P2X7 receptor on APP processing, contradicting the initial claims of a deleterious role for P2X7 receptor activation in AD.

Follow up studies using the same mouse neuroblastoma cells cited above, but applying a different methodology for quantification of APP fragments, challenged those controversial findings. Accordingly, two independent studies demonstrated that BzATP-induced P2X7 receptor activation leads to decreased α-secretase activity, which was prevented by P2X7 receptor antagonists and P2X7 receptor siRNA (León-Otegui et al., 2011; Miras-Portugal et al., 2015).

In agreement with these findings, studies performed in J20 mice, an *in vivo* model of AD in which Aβ senile plaques develop spontaneously, demonstrated decreased Aβ plaques in hippocampus of AD animals upon treatment with P2X7 receptor antagonist BBG. This effect was attributed to a shift toward APP metabolism by α-secretase via reduction of GSK3 activity (Diaz-Hernandez et al., 2012), an enzyme known to phosphorylate specific AAP domains, making it more suitable to be processed into Aβ.

Altogether, those studies suggest that modulation of microglia functions, as well as regulation of APP processing by P2X7 receptor antagonists, may be a promising therapeutic strategy in the management of AD.

### Role of P2X7 receptor in Parkinson's disease

Parkinson's disease (PD) is a motor illness clinically characterized by bradykinesia, rigidity, postural instability, and tremor at rest, caused by progressive impairment of the striatal dopaminergic system that evolves to loss of dopaminergic (DA) neurons in the substantia nigra. While the mechanisms enrolled in PD development are still not completely understood, a growing body of evidence shows that chronic neuroinflammation and excessive microglia activation strongly contribute to degeneration of DA neurons within the substantia nigra and striatum of PD patients (Durrenberger et al., 2012). Oligomerization and accumulation of α-Synuclein (ASN) is also believed to be an important player in the pathophysiology of PD. ASN mediates neurotoxicity by inducing deregulation of dopaminergic and glutamatergic neurotransmission and by activating microglia, with subsequent production of pro-inflammatory molecules and reactive oxygen species (Stefanis, 2012). Interestingly, ASN also binds and activates P2X7 receptor in microglia and this interaction appears to be key to ASN-induced microglia ROS production, as it is partially reduced in P2X7 receptor knockdown cells (Jiang et al., 2015). Additionally, ASN stimulates P2X7 receptor transcription (Jiang et al., 2015), which might explain, at least in part, the P2X7 upregulation observed in brains of patients with PD (Durrenberger et al., 2012).

*In vivo* indication of a possible involvement of P2X7 receptor in the nigrostriatal degeneration first appeared in studies using the 6-OHDA rat model of PD. In this animal model, destruction of DA neurons is induced by unilateral intranigral injection of a DA selective neurotoxin 6-hydroxydopamine (6-OHDA). This leads to marked reduction of dopamine levels and nigrostriatal lesions that are accompanied by microgliosis, as typically found in autopsy of PD patients, culminating in the development of motor symptoms, closely mimicking the human disease. Administration of selective P2X7 receptor antagonists ameliorated motor and memory deficit, and significantly attenuated 6-OHDA-induced microgliosis (Marcellino et al., 2010; Carmo et al., 2014; Ferrazoli et al., 2017; Kumar et al., 2017), suggesting a pro-inflammatory role for P2X7 receptor signaling via microglia activation in PD. In addition, P2X7 receptor antagonists significantly ameliorated 6-OHDA-induced decrease in mitochondrial respiration, as well as increase in oxidative stress and mitochondrial-linked apoptosis, adding preservation of mitochondrial function and integrity to the protective mechanisms of P2X7 receptor antagonists in PD (Kumar et al., 2017).

While the evidence supporting the involvement of P2X7 receptor in PD neuroinflammation is strong, whether direct activation of neuronal P2X7 receptor is responsible for PD neurodegeneration has been subject of discussion. Sensitivity of dopaminergic neurons to ATP- and 6-OHDA-induced cytotoxicity via activation of P2X7 receptor has been demonstrated *in vitro* (Jun et al., 2007; Carmo et al., 2014). Along the same lines, *in vitro* studies performed in neuroblastoma SH-SY5Y cells demonstrated that extracellular ASN directly activates P2X7 receptor to induced ATP release, contributing to neuronal toxicity in PD (Wilkaniec et al., 2017). However, the association between activation of neuronal P2X7 receptor and DA neuronal loss *in vivo* remains to be proven. Intranigral injection of P2X7 receptor antagonist A438079 attenuated depletion of striatal dopamine stores but it did not affect loss of dopaminergic cells in the 6-OHDA PD model (Marcellino et al., 2010). Similarly, genetic deletion and pharmacologic inhibition of P2X7 receptor failed to promote neuroprotection in a mouse model of PD induced by administration of the dopaminergic neurotoxin MPTP (Hracskó et al., 2011).

On the other hand, BBG treatment significantly prevented and even reversed loss of DA neurons in 6-OHDA-treated animals, as evaluated by striatal content of tyrosine hydroxylase (TH) (Carmo et al., 2014; Ferrazoli et al., 2017), the limiting enzyme in the production of dopamine often used a surrogate for number of DA neurons. Additional work done in an endotoxin-induced model of PD confirmed the neuroprotective properties of BBG. As in the rat 6-OHDA model, brain of LPS-treated mice showed high levels of P2X7 receptor expressing activated microglia in the substantia nigra that was, once again, reduced by selective inhibition of the P2X7 receptor by BBG. Lower microglia activation correlated with marked protection of DA neurons from LPS-induced cytotoxicity, as assessed by number of TH^+^ cells (Wang et al., 2017).

Collectively, those studies strongly support the hypothesis that P2X7-induced microglia activation is involved in PD pathology. However, they fail to demonstrate whether neuroprotection conferred by P2X7 receptor antagonistic results solely from inhibition of microgliosis or from direct effect on neuronal P2X7 receptor. Using the current immunohistochemistry tools, P2X7 receptor expression was showed to co-localize with nigrostriatal glia in animal models of PD, not neurons. Brain analysis of the recently generate, conditional humanized P2X7 receptor mice confirmed the expression of P2X7 receptor in all major non-neuronal lineages throughout the brain (astrocytes, microglia and oligodendrocytes), as well as glutamatergic pyramidal neurons of the hippocampus (Metzger et al., 2017), further questioning the existence of these receptors in DA neurons.

### Role of P2X7 receptor in multiple sclerosis (MS)

Multiple sclerosis (MS) is an immune-mediated, chronic degenerative disease of the CNS, characterized by inflammatory focal lesions in both white and gray matter, immune cell infiltration, loss of oligodendrocytes, and axonal damage, leading to demyelinization and neuronal death. Clinically, patients present neurological deficit including sensory disturbances, lack of motor coordination, and visual impairment (Lucchinetti et al., 2011). An autoimmune inflammatory reaction to myelin is believed to be the triggering event in the pathogenesis of MS, with genetic and environmental factors strongly contributing to disease susceptibility (Kidd, 2001; Stys, 2010). Due to the highly inflammatory nature of MS, a link between P2X7 receptor activation and MS development has been proposed.

While MS is seen most traditionally as a “white matter” disease, with loss of oligodendrocytes and damage to myelin being the core of its pathology, the involvement of other cell types such as neurons and glia on the development of this disease has also been recognized. In fact, increased in P2X7 receptor expression has been reported in oligodendrocytes, neurons and glia of MS patients.

Early post mortem studies have revealed high P2X7 expression in astrocytes and activated microglia in the brain and spinal cord of MS patients (Narcisse et al., 2005; Yiangou et al., 2006). Deregulated P2X7 expression seems to precede the symptomatology of the disease, as increased P2X7 levels are present in normal appearing optic nerve samples of MS patients (Matute et al., 2007), suggesting that deregulated P2X7 signaling might be a risk factor associated with early lesion development of MS.

A better understanding of the kinetics of P2X7 receptor expression in MS is provided by studies performed with rat models of experimental autoimmune encephalomyelitis (EAE). EAE is the most common rodent model used to study the molecular mechanisms responsible for the development of inflammation and progressive demyelination characteristics of MS (Tsunoda and Fujinami, 1996). In this model, EAE symptoms start 7 days following immunization. Full-blown symptomatology, characterized by paralysis of tail, hind limbs, and loss of reflexes, develops at 10 days. The disease spontaneously resolves by 15 days following initial immunization.

Temporal analysis of P2X7 receptor expression and its relation to disease progression showed biphasic increase on P2X7 receptor expression in the brain of EAE rats, which was most evident in the frontal motor and somatosensory cortical areas, with higher intensity in the deep cortical layers. Upregulation of P2X7 receptor occurred in astroglia cells at very early stage, when animals were still asymptomatic (Grygorowicz et al., 2010). P2X7 receptor upregulation in neurons and oligodentrocytes occurred later, corresponding to a moment of maximal neurological symptoms, and then decreases to base line levels during the recovery phase (Matute et al., 2007).

Such kinetics indicates that P2X7 receptor-induced cytokine release by glia likely contributes to the inflammatory milieu established very early in the disease development. Higher expression of P2X7 receptor in neurons and olygodendrocytes in the acute phase of the disease, corresponding to a peak in inflammatory cytokine levels and cells death, further intensifies the cytotoxic effects of P2X7 receptor signaling in these cells, contributing to progressive inflammation and degeneration in EAE. Interestingly, total P2X7 receptor levels remained high in the brain of EAE rats even 20 days after immunization and it correlates to increased levels of glial fibrillary acidic protein (GAFP), a marker of astrocyte activation, suggesting a role for P2X7 receptor activation in the sustained astrocytosis that is observed in the advanced stages of the disease, in both EAE and MS, despite clinical recovery (Holley et al., 2003; Grygorowicz et al., 2011). A study performed in brain samples from MS patients has shown up-regulation of P2X7 receptor on astrocytes in the parenchyma of frontal cortex from secondary progressive MS patients, validating the animal findings (Amadio et al., 2017).

Increased P2X7 receptor expression correlates with higher P2X7 receptor signaling in MS. Supporting this assumption, *in vitro* and *in vivo* animal studies have shown that enhanced ATP signaling via P2X7 receptor activation leads to oligodendrocytes excitotoxicity and death, resulting in tissue damage that highly resembles MS lesions. Moreover, administration of P2X7 receptor antagonist to EAE rats significantly decreases astrogliosis, reduces demyelination, and improves neurological symptoms (Matute et al., 2007; Grygorowicz et al., 2016). These findings reinforce eATP-P2X7 signaling as a key factor contributing to MS pathology and the potential use of P2X7 pharmacological blockers to prevent or ameliorate MS symptoms.

Interestingly, temporal analysis of P2X7 receptor expression and activity in myeloid cells in MS patients demonstrated that P2X7 receptor is present but inhibited on peripheral monocytes during the acute phase, while totally absent from microglia/macrophages during the secondary progressive phase. This possibly granted these cells increased survival and further contributed to neuroinflammation in both phases of the disease (Amadio et al., 2017).

Several independent European and Australasian case-control cohort discovery studies have identified a significant association of a rare, loss-of-function, minor allele frequency P2X7 receptor single nucleotide gene polymorphism (SNP), rs28360457, coding for Arg307Gln with protection against MS. The same European cohort also demonstrated a significant association of gain-of-function haplotype in P2X7 receptor with increased risk of MS. Functional studies using monocytes of normal subjects heterozygous carriers of rs28360457 revealed dominant negative effects on pore formation and downstream pro-inflammatory effects, while monocytes harboring the gain-of-function haplotype showed receptor activity two-fold higher than controls (Gu et al., 2015). In addition, a Spanish case-control association study found increased frequency of a gain-of-function SNP, rs17525809, in MS patients compared to control (Oyanguren-Desez et al., 2011). Altogether, the mentioned studies strengthen the hypothesis that P2X7 receptor's gene variants may play a major role in the pathogenesis of MS. Such data also reinforce P2X7 receptor increased expression and functionality as an important feature in MS disease.

### Role of P2X7 receptor in Huntington's disease (HD)

Huntington's disease is an inherited neurodegenerative disorder caused by a CAG triplet-repeat expansion coding for a polyglutamine (polyQ) sequence in the N-terminal region of the huntingtin (HTT) protein, leading to progressive degeneration of nerve cells in the brain, predominantly in the cortex and striatum. Clinically, patients present progressive cognitive decline, motor, and psychological dysfunction. The molecular mechanisms behind HTT toxicity are still under investigation, but recent studies have suggested a possible role for ATP and P2X7 receptor activation in the pathophysiology of HD.

Increased mRNA and protein levels of P2X7 receptor were described in the brain of two distinct genetic mouse models of HD (Tet/HD94 and R6/1) as compared to WT mice (Díaz-Hernández et al., 2009). In both animal models, P2X7 receptor protein levels were increased at the onset of the motor impairment, and occurred in axonal processes of cortical neurons projecting to the striatum, particularly at the level of synaptic terminals. Moreover, aging related decline of P2X7 receptor levels in striatum that normally occurs in WT mice was less prominent or totally absent in HD mice. Increased synaptic P2X7 receptor expression correlated with modified calcium flux in response to BzATP in synaptosomes isolated from HD mice vs. control WT, suggesting altered P2X7 function in HD synaptic terminal. This may explain the increased sensitivity to BzATP-induced apoptosis in mutant-HTT-expressing primary cortical neurons in culture, which was prevented by BBG (Díaz-Hernández et al., 2009). However, how mutant HTT expression modulates P2X7 receptor levels remains unknown.

Supporting evidence for the involvement of P2X7 receptor in HD onset and development comes from the fact that BBG treatment prevented body weight loss and deterioration of motor coordination of R6/1 mouse model of HD as compared to vehicle-treated HD mice.This protective effect might be correlated with lower caspase-3 activation in neurons (Díaz-Hernández et al., 2009). Interestingly, P2X7 receptor inhibition was only beneficial when BBG was administered at the age that immediately preceded symptom onset, and had no significant effect when used at earlier ages. This data suggests that a therapy using P2X7 antagonists might be successful only when administered at more advanced stages of the disease, when P2X7 receptor expression is already altered. An important note, despite several reports of the anti-inflammatory effects of P2X7 receptor antagonists in microglia, BBG did not affect reactive gliosis in this HD model (Díaz-Hernández et al., 2009).

Collectively, all those studies support the hypothesis that upregulated expression and function of P2X7 receptor contribute to the pathogenesis of several neuroinflammatory and neurodegenerative diseases, and identify P2X7 receptor as a potential druggable therapeutic target.

## Brain permeable P2X7 receptor pharmacological inhibitors

The development of CNS-permeable P2X7 receptor antagonists has been at the center of intense efforts in the pharmaceutical industry in the last 15 years, a task that has been quite challenging due to poor molecular stability and pharmacokinetic properties. Availability of P2X7 ligands and inhibitors suitable for *in vivo* studies is limited. Despite that, several *in vivo* studies have shown benefits of P2X7 receptor antagonists in animal models of neurodenegerative diseases.

BBG is the P2X7 antagonist most used in animal models of brain disease due to its ability to efficiently cross the blood-brain-barrier (BBB) and infiltrate the brain parenchyma. Intraperitoneal administration of 54.5 mg/kg of BBG results in 200 nM of this compound in the brain in mice, a concentration that is within the range of IC_50_ of BBG to antagonize P2X7 receptor (10–200 nM) and bellow the DL50 by one order magnitude. BBG efficiency in improving disease outcomes has been repeatedly reported in mice models of HD, AD, PD, and epilepsy. However, it is less efficient at antagonizing P2X7 receptor in human than rodents (Jiang et al., 2000). In addition to BBG, the P2X7 receptor blocker A438079 can also cross the BBB, but its use in animal studies has been restricted due to its short half-life (1.02 h) and limited bioavailability (19%) (Nelson et al., 2006). Such pharmacokinetic properties make A438079 inappropriate for chronic applications or clinical purposes. (Díaz-Hernández et al., 2009; Marcellino et al., 2010).

While several classes of specific P2X7 receptor inhibitors have been developed lately, only few of them can efficiently cross the BBB. Despite this progress, finding a potent, selective and CNS penetrable P2X7 receptor inhibitor with acceptable pharmacokinetics (PK), particularly clearance, is still challenging academic researchers and pharmaceutical companies.

In 2010, Pfizer developed a potent P2X7 receptor antagonist (IC_50_ 27 nM), 2-chloro-N-((4,4-difluoro-1-hydroxycyclohexyl)methyl)-5-(5-fluoropyrimidin-2-yl)benzamide, which is an improved version of 2-chlorobenzamide template previously disclosed by AstraZeneca (Guile et al., 2009). This compound showed excellent CNS penetrability, with a brain/plasma ratio of 1.3, in addition to better PK properties when tested in rats: clearance of 3.3 mL/min/kg, 4.6 h half-life and high oral bioavailability of 84% (Chen et al., 2010).

Three years later, Janssen Research and Development released preclinical studies using two newly developed P2X7 receptor antagonists (Letavic et al., 2013). Both compounds demonstrated high potency across different species and reached high levels in the animal's brain. They were also able to successfully reduce signs of neuroinflammation, epilepsy, and seizure, as well as improve behavior in animal models of schizophrenia, hyperactivity, and social stress. However, their compounds had poor PK properties, making them unsuited to further clinical applications (Letavic et al., 2017).

Researchers from The University of Sydney and University of South Australia have recently discovered a new adamantly-cyanoguanidine hybrid compound (O'Brien-Brown et al., 2017). This new hybrid molecule combined two promising classes of P2X7 receptor antagonist as to retain desirable characteristics of each category, i.e., good pharmacokinetic properties of truncated cyanoguanidines scaffold reported by Abbott Laboratories and high potency and selectivity of the adamantyl amide moiety disclosed by AstraZeneca. Using structure-activity studies (SAR) and *in vitro* measurement of P2X7 receptor activity, they identified two promising P2X7 receptor antagonist candidates (IC50 < 50 nM). Unfortunately, the compound identified as having the best druggability, a 3-pyridyl analog, showed poor PK in mice, with a fast clearance (117 mL/min.kg) and extremely short half-life (0.22 h), making it unsuitable for human clinical studies (O'Brien-Brown et al., 2017). While brain levels of the compound were not measured, indirect evidence for CNS penetrability and inhibition of P2X7 receptor *in vivo* was provided by improvement in scape-oriented behavior in forced swimming test (FST), a test designed to evaluate depressive behavior in mice, following administration of the compound. This observation agrees with previous FST studies performed in P2X7 KO mice (Boucher et al., 2011).

Janssen Research & Development has also been working on a series of modification substitutions of the 4-methyl-6,7-dihydro-4H-triazolo[4,5-c] pyridine core in order to develop a P2X7 receptor antagonist suitable for clinical use (Letavic et al., 2017). *In vivo* studies in rats and dogs using the most promising candidate among those modified molecules have shown efficient inhibition of IL-1β release in the brain, with no significant clinical or metabolic side-effects even at the highest administered doses. Based on such performance on PK, efficacy and safety test in animals, Jansen has moved to its first steps in clinical development of their compound and safety tests in healthy volunteers are on their way (Letavic et al., 2017). Their newly developed compound demonstrated very low clearance across several species, including non-human primates, and high bioavailability, which predicted human clearance of 0.6 mL/kg and half-life of 29 h.

Despite all advances in the development of CNS penetrable P2X7 receptor antagonist and promising preclinical evidence, no clinical trial using P2X7 antagonists for treatment CNS disorders have been conducted to date. GlaxoSmithKline completed a phase I clinical trial to evaluate safety and pharmacodynamics of its pyroglutamate-based P2X7 inhibitor GSK1482160, intended for the treatment of pain and inflammation. Interestingly, this compound has good CNS penetration (B/P ~0.5). However, while no major safety issues were observed, conclusions from phase I clinical trial led to the discontinuation of the study (Ali et al., 2013). In fact, only three other P2X7 receptor antagonists have been used in clinical trials. In 2009, Pfizer completed a Phase 2a, Randomized, Double-Blind, Placebo-Controlled clinical trial to evaluate the compound CE-224,535 for the treatment of signs and symptoms of rheumatoid arthritis (Stock et al., 2012). Few years later, AstraZeneca performed a phase IIa study and subsequently in a phase IIb clinical trial to assess the effects of AZD9056 on the symptoms of rheumatoid arthritis as well. Unfortunately, despite encouraging results regarding safety, all trial failed to demonstrate efficacy of the compounds in the treatment of RA (Keystone et al., 2012).

## Conclusion

P2X7 is a multifaceted receptor involved in several physiological and pathological conditions in different body systems by modulating cellular responses in immune and non-immune cells. In infectious inflammatory disease and cancer, P2X7 receptor can have different and contrasting effects, being an angel or a demon depending on its level of activation, cell studied, and type of pathogen.

Overall, ATP-P2X7 receptor signaling boosts the immune system in the context of pathogen infections, activates microbicidal mechanisms, and induces the production of inflammatory mediators in phagocytic cells, as well as modulates adaptive immune responses. Nevertheless, P2X7 receptor activation can generate both protective and deleterious responses depending on the type of pathogen, virulence, and severity of infection. The activation of this ATP-gated ion channel shows deleterious effects during infections by hypervirulent pathogens, inducing severe inflammation and necrotic cell death, release of large amounts of ATP, which results in sustained P2X7 receptor activation, leading to a self-sustained pro-inflammatory cycle (Figure 1).

**Figure 2:**
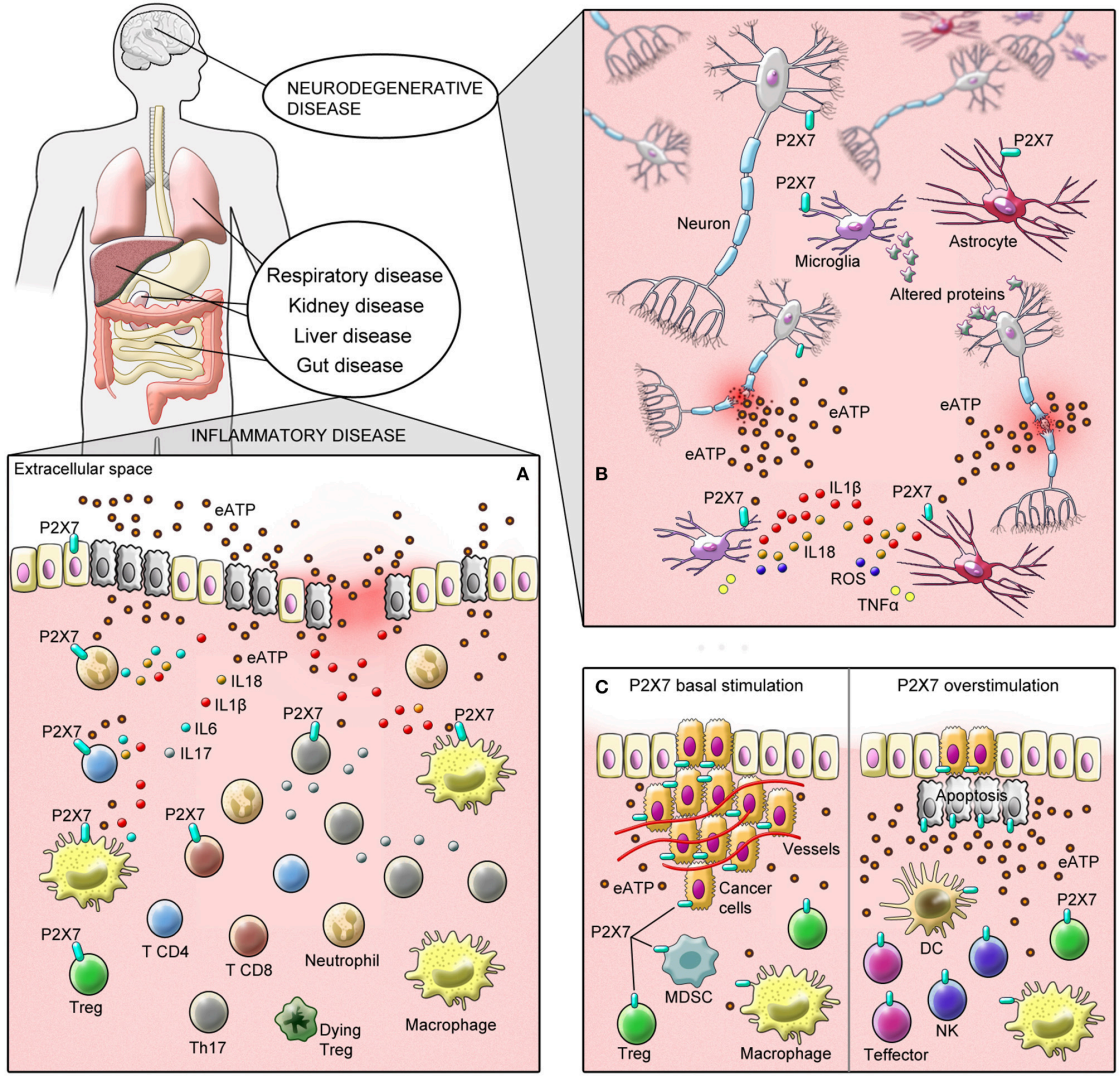
Schematic illustration showing P2X7 receptor deleterious (demon) effects in inflammatory and neurodegenerative disease. **(A)** ATP can be released from injured immune and non-immune cells activating P2X7 receptor. As a consequence, P2X7 receptor activation induces large-scale ATP release—chiefly via pannexin hemichannels—acting as a start point to inflammation. In this scenario, P2X7 activation contributes to the pathogenesis of several inflammatory diseases by activating intracellular signaling pathways and stimulating the production of pro-inflammatory mediators (i.e., ROS, NO, chemokines, and cytokines). In addition, P2X7 receptor induces T cell activation, decreases the suppressive activity and viability of Treg cells and favors the polarization of T cells into Th17 cells promoting inflammation and cell death. **(B)** Likewise, microglial P2X7 receptor activation in neurodegenerative diseases induces inflammasome activation and release of pro-inflammatory cytokines and ROS, leading to the activation of NF-κB signaling, upregulation of pro-inflammatory and pro-apoptotic genes, and death of surrounding cells, including neurons. ATP released from dying cells perpetuates the inflammatory and degenerative cycle, at least in part, via P2X7 receptor. **(C)** P2X7 receptor activation has been linked to conflicting effects on carcinogenesis, being able to exert either pro-tumoral (demon) or anti-tumoral (angel) effects depending on its level of activation and cell type studied. Left: Pro-tumoral effects are associated with P2X7 receptor basal stimulation in tumor cells as well as its activation in immunussupresive cells such as MDSCs and M2-macrophages. Right: On the other hand, anti-tumoral effects can be trigger by P2X7 overstimulation with high levels of exogenous ATP in tumor cells, resulting in membrane pore formation and cell death through apoptosis. In this inflammatory context, eATP accumulation also activates P2X7 receptor highly expressed in DCs, T effectors, NK, and macrophages, boosting the immune response against tumors.

Likewise, increased expression and prolonged activation of P2X7 receptor contribute to the pathogenesis of several inflammatory and neurodegenerative diseases. In the context of these pathologies, P2X7 receptor acts as a start point to initiate inflammation and promotes disease progression by regulating T cell adaptive immune responses. Initially, it can be activated by ATP released from both injured immune and non-immune cells. As a consequence, P2X7 induces large-scale ATP release, boosting inflammation and purinergic signaling. Upon activation, P2X7 triggers intracellular signaling pathways, stimulates the production of pro-inflammatory mediators (i.e., ROS, NO, chemokines, and cytokines), and, consequently, promotes the migration of immune cells to the inflammatory site. In addition, P2X7 receptor induces T cell activation, decreases the suppressive activity and viability of Treg cells and favors the polarization of T cells into Th17 cells, promoting inflammation and cell death (Figure 2). Therefore, P2X7 receptor pharmacological inhibition or genetic deletion in different inflammatory models completely abrogates or significantly attenuates inflammation and disease progression.

Finally, the outcomes of studies—aimed at understanding P2X7 pharmacology and using P2X7 receptor agonists, antagonists, and P2X7 KO mice—should be carefully analyzed and discussed in order to accurately bring up the biology and relevance of this receptor in the context of inflammatory and infectious diseases. In addition, peculiarities of each parasite infection and inflammatory disease should be considered when developing pharmacological treatments targeting P2X7 receptor.

## Author contributions

LS, PdA, CdS, and RC-S wrote the article. All authors contributed to the study conception and design, and critically revised the manuscript.

### Conflict of interest statement

The authors declare that the research was conducted in the absence of any commercial or financial relationships that could be construed as a potential conflict of interest.
